# Association of Climatic Variability, Vector Population and Malarial Disease in District of Visakhapatnam, India: A Modeling and Prediction Analysis

**DOI:** 10.1371/journal.pone.0128377

**Published:** 2015-06-25

**Authors:** Ravi Chandra Pavan Kumar Srimath-Tirumula-Peddinti, Nageswara Rao Reddy Neelapu, Naresh Sidagam

**Affiliations:** 1 Department of Biochemistry and Bioinformatics, GITAM Institute of Science, GITAM University, Rushikonda Campus, Visakhapatnam, Andhra Pradesh, India; 2 Department of Statistics, College of Science and Technology, Andhra University, Waltair, Visakhapatnam, Andhra Pradesh, India; University of New England, AUSTRALIA

## Abstract

**Background:**

Malarial incidence, severity, dynamics and distribution of malaria are strongly determined by climatic factors, i.e., temperature, precipitation, and relative humidity. The objectives of the current study were to analyse and model the relationships among climate, vector and malaria disease in district of Visakhapatnam, India to understand malaria transmission mechanism (MTM).

**Methodology:**

Epidemiological, vector and climate data were analysed for the years 2005 to 2011 in Visakhapatnam to understand the magnitude, trends and seasonal patterns of the malarial disease. Statistical software MINITAB ver. 14 was used for performing correlation, linear and multiple regression analysis.

**Results/Findings:**

Perennial malaria disease incidence and mosquito population was observed in the district of Visakhapatnam with peaks in seasons. All the climatic variables have a significant influence on disease incidence as well as on mosquito populations. Correlation coefficient analysis, seasonal index and seasonal analysis demonstrated significant relationships among climatic factors, mosquito population and malaria disease incidence in the district of Visakhapatnam, India. Multiple regression and ARIMA (I) models are best suited models for modeling and prediction of disease incidences and mosquito population. Predicted values of average temperature, mosquito population and malarial cases increased along with the year. Developed MTM algorithm observed a major MTM cycle following the June to August rains and occurring between June to September and minor MTM cycles following March to April rains and occurring between March to April in the district of Visakhapatnam. Fluctuations in climatic factors favored an increase in mosquito populations and thereby increasing the number of malarial cases. Rainfall, temperatures (20°C to 33°C) and humidity (66% to 81%) maintained a warmer, wetter climate for mosquito growth, parasite development and malaria transmission.

**Conclusions/Significance:**

Changes in climatic factors influence malaria directly by modifying the behaviour and geographical distribution of vectors and by changing the length of the life cycle of the parasite.

## Introduction

Malaria is a serious concern in most of the developing countries including India. It was estimated that by the year 2020, 36% of the world population living in tropical and sub-tropical regions of 107 countries would be affected by malaria. India is among those eleven countries in South Asia region, where 1.2 billion (85.7%) of the population is exposed to the risk of malaria [1] and Visakhapatnam is one of the major districts in the State of Andhra Pradesh receiving highest incidences of malaria [2, 3]. Statistical analysis, modelling of parameters, mapping of disease density and identifying the mechanism of transmission in endemic areas of malaria are the different approaches used to provide insights on malaria disease. Prevalent malarial disease data was collated to map the intensity and density of disease and also to identify the malarial risk zones at continent, country and regional level [4]. A malarial atlas project with a goal of developing a global malarial map provided insights on global endemic patterns, and areas with inadequate data on endemicity [5–7]. Malaria disease maps can also be used for targeted control and monitoring of the progression of disease. In most of the situations *Plasmodium falciparum* endemicity was used to develop malarial maps. Till date, there were no maps of malaria in the district of Visakhapatnam. Therefore, there is a great need to map total malaria disease, *P*. *falciparum* and *P*. *vivax* endemicity in the district of Visakhapatnam.

Climatic factors such as temperature, rainfall and relative humidity and non-climatic factors such as parasites, vectors, human host factors, population movement or migration, urbanization and interruption of control and preventive measures favor the transmission of the disease [3]. Malaria disease incidence, severity and distribution are closely related to naturally existing environmental conditions [8], climatic factors [9], disease carrying vectors [10] and human activities [11]. Dynamics of the distribution of malaria and estimates indicate that 90% of malarial cases are strongly determined by environmental factors and climatic factors [8, 12]. The three main climatic factors that affect malaria are temperature, rainfall, and relative humidity [13]. Malarial vector distribution, behaviour and life cycle of the parasite are also influenced by changes in temperature, rainfall, and relative humidity [14–16]. Therefore, determining the changes in temperature, rainfall and humidity of Visakhapatnam that can influence malarial vector and life cycle of a parasite is of paramount importance.

Statistical analysis has an important role in various kinds of public health and epidemiological studies. Application of statistical analysis helps us in understanding the geographical distribution of the incidence of a malaria disease, and its relationship to potential risk factors. Relationship between the climatic factors and malaria was studied in Ghana [17], Ethiopia [18, 19], Zimbabwe [20], Tanzania [21, 22], Sri Lanka [23], China [24, 25], Uganda [26], Kenya [27], Sudan [28], Rwanda [29], Madagascar [30], Tibet [31], Ghana [32] whereas limited studies are carried out in malarial endemic areas Dehradun of Uttaranchal, [33], Sonitpur [34] and Kokrajhar of Assam [35] in India. Relationship between the climatic factors, mosquito population was studied in Bangladesh [36], California [37], Tanzania [38], Ghana [32], Saudi Arabia [39], West Africa and Europe [40], Nigeria [41] Italy [42] and around the world [43], whereas limited studies are carried out in malarial endemic areas Dehradun [33] and Varanasi [44] of India. Though the relationship between climatic factors, malaria disease and mosquito population was well studied in different countries and India, there were meagre or no reports on similar kind of studies in the district of Visakhapatnam. Therefore, a study to determine the relationship between climatic factors, malaria disease and mosquito population in the district of Visakhapatnam is essential.

Malaria is a seasonal dependent disease where seasonal indexes of malaria disease, mosquito population and climatic factors data were estimated and predicted to understand the effect of seasons on malaria disease [20, 35, 45–52], demonstrated impact of season on malaria, whereas lack of seasonal impact was revealed by Smith et al., [53] and Mpofu [54]. Seasonal indexes of malaria disease and rainfall were well studied with due importance, whereas seasonal indexes of temperature were given less importance [55]. Though there were meagre reports of using the seasonal index in India [35], no reports were there on application of seasonal index in the district of Visakhapatnam. Therefore, a study to determine the seasonal indexes of climatic factors, malaria disease and mosquito population in the district of Visakhapatnam are indispensable.

Applying statistical models is a helpful strategy in the analysis of the available data, and describing the statistical relationship between potential risk factors and disease incidence/prevalence [56, 57]. Statistical models are used as a guideline to build models [58] based on some of the features to understand Malaria Transmission Mechanism (MTM). Regression analysis [18, 35] was used to assess the association between the variables. Multiple regression analysis [59] and Auto Regressive Integrated Moving Average (ARIMA) modeling [17, 35, 60, 61] are used to model and predict the malaria disease incidence. Thousands of malarial cases are registered every year in this district of Visakhapatnam and meagre consolidated available data on the disease demands a case study to understand Malaria Transmission Mechanism [3]. Thus, to improve the monitoring activities of the disease, statistical models can be used as supplementary to understand malaria. However, to date, no case studies were done in the district of Visakhapatnam to model and predict climatic factors, mosquito population and malaria disease incidence. Hence, the objectives of the current study are mapping of malaria disease incidence cases; evaluating statistical relationships; modeling and predicting climatic factors, mosquito population and malaria disease incidence; and developing an algorithm for identifying malaria transmission patterns to understand Malaria Transmission Mechanism in the district of Visakhapatnam, India.

## Materials and Methods

### Study area

**Fig 1:**
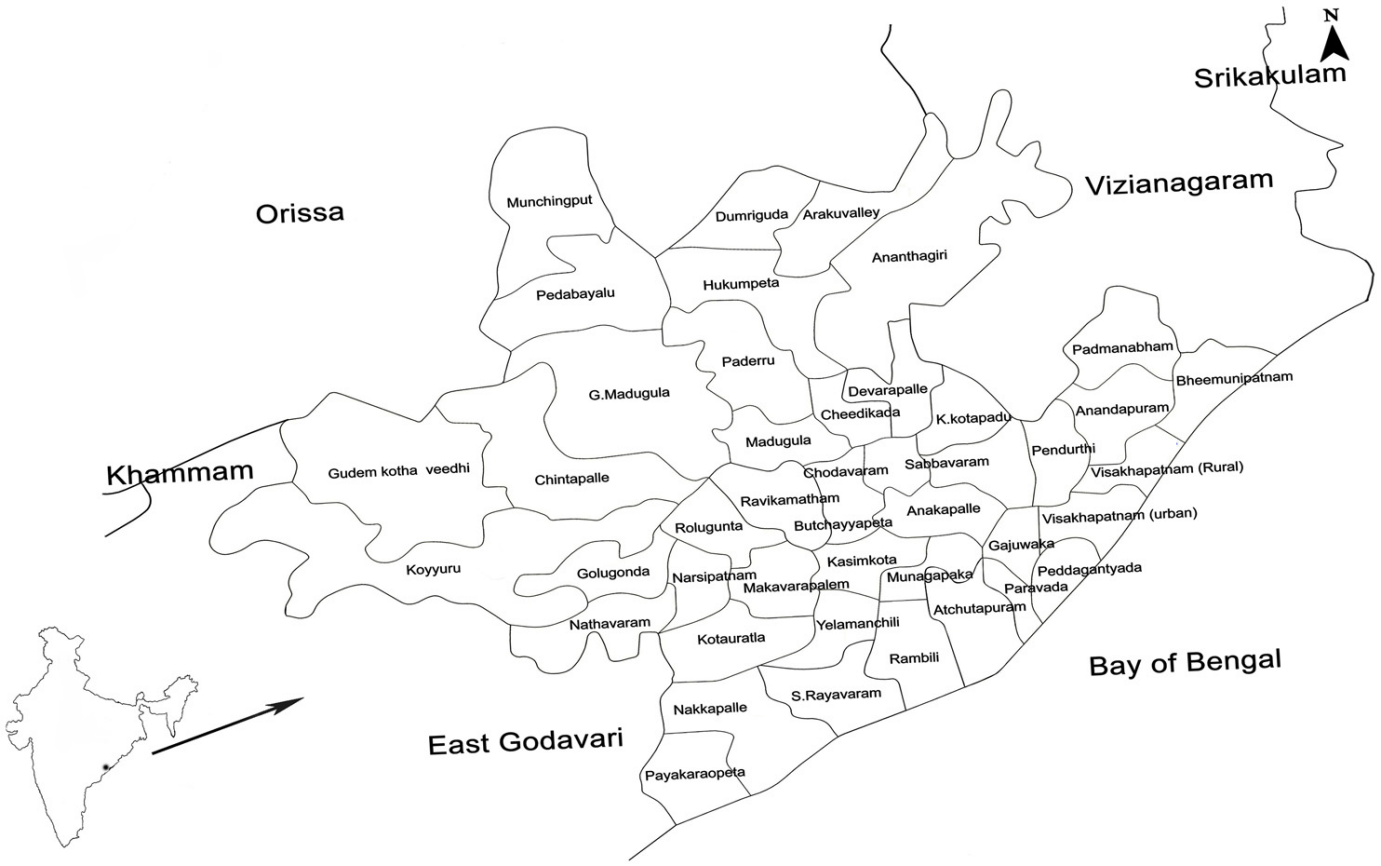
Map of the study area Visakhapatnam district showing the total mandals.

Visakhapatnam district stands in 44th place in the country and 5^th^ in Andhra Pradesh state in terms of populations and a geographical area of 11.24 lakhs hectares with long sea coast line (Fig 1). Visakhapatnam the north coastal district of Andhra Pradesh is located between 17°15' and 18°32' North latitude and 18°54' and 83°30' east longitude. It is bounded in the north, partly by Orissa and Vizianagaram district, in the south of East Godavari district, in the west of Orissa and in the east of the Bay of Bengal. According to the 2011 India census, population of Visakhapatnam is 37,89,820 with an area covering about 11,161 square kilometers. The coastal regions are pleasantly humid and comfortable, further inland the air gets warmer while in hill areas, it is noticeably cooler on account of elevation and vegetation with little variation in temperature through the year. May is the hottest month with average temperatures around 32°C (90°F), while January is the coolest month with average temperatures near 23°C (73°F). The humidity remains high throughout the year. The total annual rainfall is around 945mm (38inches), the bulk of which is received during the south-west monsoon. October is the wettest month with around 204mm (8 inches) of rainfall [3]. Visakhapatnam district has 43 mandals for effective administration and 86 primary health centres for effective medical services to the public (Fig 1).

### Sampling of data

#### Malaria disease incidence data

*P*. *falciparum* and *P*. *vivax* are responsible for Malaria disease incidence in the district of Visakhapatnam. A monthly malaria epidemiological data related to *P*. *falciparum*, *P*. *vivax*, and total of *Plasmodium* sps. of every Primary Health Centre (P.H.C.) in each mandal in the district was obtained from National Vector Borne Disease Control Programme (N.V.B.D.C.P.) office, Visakhapatnam District for the periods of 2005–2011 (Table 1; S1 Dataset).

**Table 1:** Surveillance centres used for collection of malaria disease incidence and vector data.

Category	Surveillance Centres	
	Primary Health Centres	Vector collection centres
Urban	Visakhapatnam, Anakapalli [urban], A. M. Unit Steel Plant	GVMC Visakhapatnam, GVMC RHC Simhachalam, GVMC Vadlapudi, GVMC A.M Unit Gajuwaka, Municipality Anakapalli
Rural	Ananthagiri, Bhimavaram, Pinakota, Lungaparthy, Gannela, Madagada, Dumbriguda, Killoguda, Hukumpeta, Uppa, Minumuluru, Edulapalem, Pedabailu, Gomangi, Rudakota, Munchingput, Labburu, G.Madugula, Gammeli, Lothugedda, Korukonda, Lambasingi, Tajangi, G.K.Veedhi, Jerrila, Pedavalasa, Darakonda, Sapparla, Downuru, K.D.Peta, Kantaram, R.J.Palem, U.Cheedipalem	Satyavaram, Nakkapalli, Munagapaka, Yelamanchilli
Tribal	Nathavaram, Kasimkota, Thallapalem, Thummapala, Munagapaka, Chuchukonda, Sabbavaram, Gullepalli, Gajuwaka, Vadlapudi, Parawada, V.Cheepurapalli, Revidi, R.Thallavalasa, Anadhapuram, Pendurthi, Madurawada, Chowduwada, L.V.Palem, Gavaravaram, Thurakalapudi, Butchaipeta, Vaddadi, Ravikamatham, Devarapalli, Vechalam, Payakaraopeta, Sreerampuram, Godicherla, Sravasiddi, Penugollu, Regupalem, Rambilli, Dimili, Atchutapuram, Haripalem, Makavarapalem, Kotauratla, Vemulapudi, Rolugunta, K.J.Puram, Cheedikada, Pedagogada, Golugonda	A.A.Giri, Gennela, Madagada, Minumuluru, Pedabayulu, KD Peta, R.J.Palem

#### Mosquito population data

Four main vectors which are responsible for malaria transmission in the district of Visakhapatnam are *Anopheles culicifacies*, *A*. *stephenis*, *A*. *annularis* and *A*. *fluviatilis*. *A*. *culicifacies* and *A*. *stephenis* are the major mosquitoes in rural and urban areas respectively. *A*. *fluviatilis* and *A*. *annularis* are the primary and secondary mosquitoes for malaria transmission respectively. A monthly mosquito population data of all the mosquitoes and vector stations was obtained from National Vector Borne Disease Control Programme (N.V.B.D.C.P.) office, Visakhapatnam District for the periods of 2006–2011 (Table 1; S2 Dataset).

#### Climatic factors data

A monthly total rainfall, monthly average minimum and maximum temperatures, average relative humidity—RH1 (8:00 hrs) & RH2 (2:00 hrs) for the period 2005–2011 of Visakhapatnam district was procured from Indian Meteorological Department (IMD) and the Cyclone Warning Centre, Visakhapatnam (S3 Dataset).

### Data analysis

Data was analysed to map malaria disease; evaluate the statistical relationship of climatic factors, mosquito population and malaria disease incidence; model and predict climatic factors, mosquito population and malaria disease incidence; and in identifying malaria transmission patterns to understand Malaria Transmission Mechanism in the district of Visakhapatnam.

#### Mapping of malarial disease and vector population stations

Malaria disease incidences were aggregated by P.H.C. by mandal by month by year. Aggregated malaria disease incidences for 7 years from 2005–2011 was taken to map the malaria disease incidences in each mandal. Infected cases of total human population for 7 years from 2005–2011 was taken to calculate the average percentage and was sorted from highest to lowest disease incidences in each mandal. This data was used to develop three maps—map representing total disease incidence, map representing disease incidence due to *P*. *falciparum* and map representing disease incidence due to *P*. *vivax*. Mosquito population data was available for 6 years from 2006–2011. The mosquito collection, field stations in Visakhapatnam were also mapped.

#### Evaluating statistical relationships of climatic factors, mosquito populations and malarial disease incidence

Magnitude, trends, seasonal indexes of malaria disease incidences, mosquito population, relative humidity, rainfall and also the correlation among these factors are evaluated.

#### Magnitude of malaria

Annual fluctuations of malaria disease incidence was carried out by using malarial cases as epidemiological indicators in order to understand the magnitude of the problem and to explore the trends of malaria cases in the district of Visakhapatnam. In this case all the data points on malaria disease incidence were aggregated by month by year in the district. In addition, climatic factors and mosquito population were also aggregated by month by year in the district of Visakhapatnam.

#### Trends of malaria

Exploring monthly variations of malaria disease incidence, mosquito population, total rainfall, maximum temperature, minimum temperature and relative humidity would provide us a better understanding of the trends of malaria disease in the district of Visakhapatnam. In this case all the data points on climatic factors, malaria disease incidence and mosquito population were aggregated by month in the district.

#### Correlation between the climatic variables, mosquito populations and malarial disease

To establish the relationship between two variables, correlation analysis is applicable. Correlation coefficient analysis was used to observe the relationship between the climatic factors (temperature, rainfall and relative humidity) and mosquito population and thereby the impact on malaria disease incidence. In this case all the data points on climatic factors, disease incidence and mosquito population were aggregated by month in the district.

#### Seasonal pattern and index

Seasonal patterns are responsible for the epidemiological pattern of malaria transmission [18]. Seasonal fluctuations of disease incidence, seasonal total rainfall patterns, and seasonal average maximum and minimum temperatures would provide us with information on malaria transmission pattern in the district of Visakhapatnam. In addition to the above analysis, seasonal index of malaria cases, mosquito population and relative humidity were carried out to support seasonal patterns. Seasonal index [62] is calculated for Quarter 1 (January, February, March), Quarter 2 (April, May, June), Quarter -3 (July, August, September) and Quarter 4 (October, November, December) using the following formula

$$Seasonal\;index\;=\;\frac{Average\;quarter}{Total\;average\;of\;all\;quarters}\;X\;100$$

Generally in the district of Visakhapatnam we observe three seasons—winter, summer and monsoon (South West and North East). Based on the prevalent seasons in the district of Visakhapatnam we propose a new method for the calculation of seasonal index, which is appropriate and applicable to the district of Visakhapatnam. Winter = Quarter 1 (December, January, February), Summer = Quarter 2 (March, April, May), Monsoon 1 (South West) = Quarter -3 (June, July, August) and Monsoon 2 (North East) = Quarter -4 (September, October, November). Seasonal index is calculated using the following formula

$$Seasonal\;index\;=\;\frac{Average\;of\;the\;season}{Total\;average\;of\;all\;seasons}\;X\;100$$

The variance between traditional and proposed seasonal indexes was estimated using students t- test [63].

#### Modeling and predicting climatic factors, mosquito populations and malarial disease incidence

Linear regression analysis, multiple regression analysis and auto regression analysis were used to model and predict monthly malaria disease incidence and mosquito population. In this case the data of the district on malaria disease incidences, mosquito population, relative humidity, temperature, and rainfall is simply aggregated by month for each year to model and predict. In case of seasonal modeling this monthly data is aggregated into seasons as described in calculation of seasonal indexes.

#### Linear regression analysis

Linear regression analysis is used to know the percentage of variation among one independent and one dependent variable. A simple linear regression analysis is applied to each and every independent variable (climatic factor) with the dependent variable (malaria disease cases) to know the extent of variation of malaria disease cases depending on the climatic factor. Furthermore, linear regression analysis was applied between the dependent (mosquito population) and independent variables (climatic factor) to know the individual factors influencing mosquito population [64].

#### Multiple regression analysis

Multiple regression method can be used for modeling and prediction of disease incidence by assessing the relationship between the variables [64]. By this analysis we can find out which independent variable has more significant impact on the dependent variable, i.e., multiple regression analysis can be used to know the climatic factor which has mostly influenced the malaria disease incidence.

For a model with multiple predictors, the equation is–Y = a_0_+a_1_X_1_+a_2_X_2_+……..a_k_X_k_+e and the regression equations are mentioned in Table 2. The final multiple regression model for malarial cases based on monthly data contained one significant explanatory variable for the prediction of malaria prevalence i.e., minimum average temperature. The final multiple regression model for mosquito population based on monthly data contained average minimum and maximum temperatures, total rainfall, average rainfall, relative humidity 1 and 2 for prediction of the mosquito population. The final multiple regression model for malarial cases based on seasonal data contained one significant explanatory variable for the prediction of malaria prevalence i.e., minimum average temperature. The final multiple regression model for mosquito population based on seasonal data contained no significant explanatory variable for the prediction of mosquito population.

**Table 2:** Multiple regression equations for both malarial cases and mosquito populations based on monthly and seasonal data.

Data	Regressionequation	
	Malarial Cases	Mosquito Population
**Monthly**	1691 + 0.0489(Total Rainfall in Mm) -0.35(Averege Rainfall in Mm) -167(Average Maximum Temperature) +101(Average Minimum Temperature) -69.2(Average Relative Humidity 1) +85.9(Average Relative Humidity 2) +0.902(Mosquito population).	474 + 0.00955(Total Rainfall in Mm) -0.063(Averege Rainfall in Mm) +10.0(Average Minimum Temperature) +21.3(Average Maximum Temperature) -3.23(Average Relative Humidity 1) -13.4(Average Relative Humidity 2)
**Seasonal**	1691+0.0489(Total Rainfall in Mm) -0.35(Averege Rainfall in Mm) -167(Average Maximum Temperature) +101(Average Minimum Temperature) -69.2(Average Relative Humidity 1) +85.9(Average Relative Humidity 2) +0.902(Mosquito population)	510+0.0200(Total Rainfall in Mm) -0.061(Averege Rainfall in Mm) +70.8(Average Maximum Temperature) +54.4(Average Minimum Temperature) +24.7(Average Relative Humidity 1) -77.5(Average Relative Humidity 2)

Monthly mosquito population and disease incidence was predicted using the multiple regression model in percentages. Percentage malarial cases and mosquito population were calculated using the following formulas

$$Percentage\;of\;malaria\;cases\;=\;\frac{Number\;infective\;cases\;per\;month}{Total\;population\;in\;district}\;X\;100$$

$$Mosquito\;population\;=\;\frac{Number\;of\;malaria\;mosquitoes\;per\;month}{Total\;mosquito\;population\;in\;district}\;X\;100$$

Graph was plotted to observe the variance between observed and expected (predicted) malaria disease incidence and mosquito population data. Chi-square test for goodness of fit was also administered to know whether the model is the best fit for the data or not. P-values obtained are used in testing hypothesis and to either reject or fail to reject a null hypothesis.

#### Autoregressive analysis

Auto Regressive Integrated Moving Average (ARIMA) model [19, 23] is used to model and predict malaria disease incidences and mosquito population. To better understand the data or to predict future points in the series (forecasting) ARIMA model is applied to time series data. Time series data are either stationary/non stationary (fluctuating). Models when applied to data show evidence of non-stationary and can be applied to remove the non-stationary data [65]. Autocorrelation functions serve clearly to identify autoregressive function. Autocorrelation is the correlation between two values of the same variable and it is applicable to detect the non-randomness of the data. Since the data is non-stationary (fluctuating) month wise very significantly auto correlation function was used to predict the future cases of malaria prevalence. In time series analysis, the extent of the lag in an autoregressive model is identified by the partial autocorrelation function (PACF) for both monthly and seasonal data.

Autocorrelation r (k) = autocorrelation (ACF) at lag k, k = 0, 1, 2,…

$${StandarderrorofACF\;(k)\;=\;\left(\left(1+2\sum_{m-1}^{k-1}r_{m}^{2}\right)/N\right)}^{1/2};k\;=\;1,\;2\ldots \;\ldots$$

For a time series *Z*_t_, the partial autocorrelation of lag *k*, denoted *α (k)*, is the autocorrelation between *Z*_t_ and *Z*_*t+1*_ with the linear dependence of *Z*_*t+1*_through to *Z*_*t+1–1*_ removed; equivalently, it is the autocorrelation between *Z*_*t*_and *Z*_*t+k*_that is not accounted for by lags 1 to *k* − 1, inclusive.

$$\begin{array}{l} \alpha \left(1\right)\;=\;\text{Cor}\left({\text{Z}}_{t},\;{\text{Z}}_{t+1}\right)\\ \alpha \left(k\underline{k}\right)\;=\;\text{Cor}\left({\text{Z}}_{t+k\;}-\;P_{t,k\;}\left(Z_{t+k}\right),\;Z_{t\;}-\;P_{t,k\;}\left(Z_{t}\right)\right),\;\text{for}\;k\geq 2, \end{array}$$

where *P*_*t*, *k*_ denotes the projection of x onto the space spanned by *Z*_*t+1*, ………._ *Z*_*t+k-1*_Based on the partial auto correlation function, we can judge the order of the ARIMA model. Based on the ACF it is depicted that ARIMA model of order one is the best fit to the given data, then the mathematical model of ARIMA (1) can be written as

$$X_{t}\;=\;b_{0}+\;b_{1\;}X_{t-1\;}+\;e_{t}$$

Chi-square test for goodness of fit was also administered to know whether the ARIMA (1) model is the best fit for the data or not.

Multicollinearity is predictors having correlation with other predictors. To identify predictors that are highly collinear, correlation structure of the predictor variables are examined. Then each suspicious predictor is regressed with the other predictors. Variance Inflation Factors (VIF), is a measure to estimate variance of an regression coefficient, if the predictors are correlated. If the VIF < 1, there is no multicollinearity but if the VIF is > 1, predictors may be correlated. If the correlation of a predictor with other predictors is very high, predictor is eliminated from the model.

#### Development of MTM algorithm

**Fig 2:**
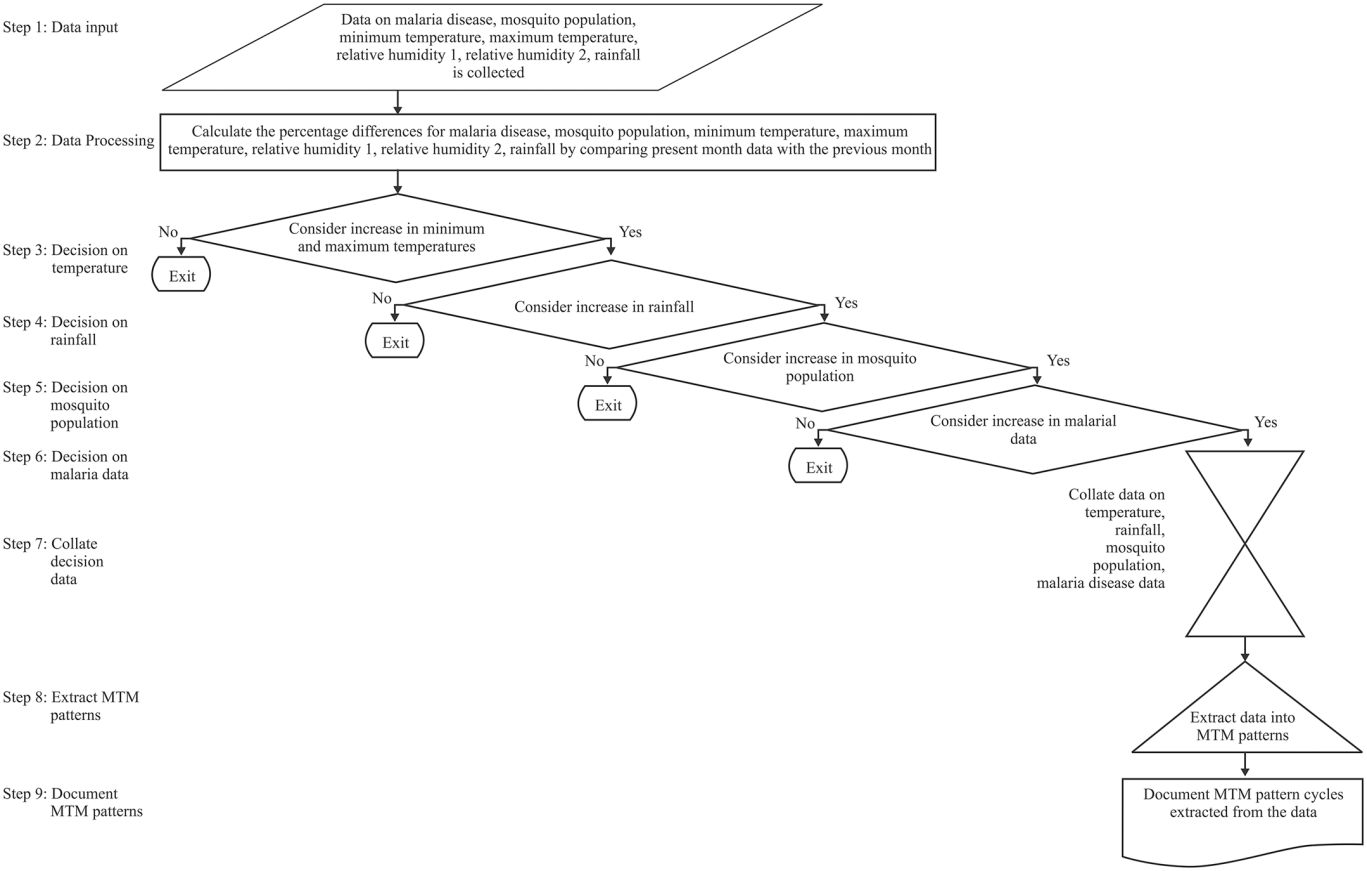
Developed malaria transmission mechanism algorithm.

Extensive literature survey provided insights on existence of malaria transmission mechanism pattern cycles that can help us to understand MTM. MTM hypothesis is that every MTM starts with fluctuations in temperature which leads to increase in rainfall. An increase in rainfall sustains the breeding sites of mosquitoes allowing to complete their life cycle, and to transmit the parasite into the host. Then the malarial parasite develops and displays signs and symptoms in the host human. A nine step MTM algorithm is developed based on the above hypothesis to identify the fluctuations in parameters leading MTM patterns (Fig 2)

Step 1 of the algorithm is Input of data: Data on malaria disease, mosquito population, minimum temperature, maximum temperature, relative humidity 1, relative humidity 2, rainfall is collected as input.

Step 2 of the algorithm is processing of data: To calculate the percentage differences in malaria disease, mosquito population, minimum temperature, maximum temperature, relative humidity 1, relative humidity 2, rainfall by comparing present monthly data with the previous month.

Step 3 of the algorithm is a decision on processed temperature data: Consider increase in minimum and maximum temperatures, if yes, continue to the next step and if no exit analysis.

Step 4 of the algorithm is a decision on processed rainfall data: Consider increase in rainfall, if yes, continue to the next step and if no exit analysis.

Step 5 of the algorithm is a decision on processed mosquito population data: Consider increase in the mosquito population, if yes, continue to the next step and if no exit analysis.

Step 6 of the algorithm is a decision on processed malaria disease data: Consider increase in malaria disease cases, if yes, continue to the next step and if no exit analysis.

Step 7 of the algorithm is collating the decision on processed data: Collate all the processed data on malaria disease, mosquito population, minimum temperature, maximum temperature, relative humidity 1, relative humidity 2, rainfall.

Step 8 of the algorithm is extracting the collated data: Extract the collated data on malaria disease, mosquito population, minimum temperature, maximum temperature, relative humidity 1, relative humidity 2, rainfall into MTM pattern cycle.

Step 9 of the algorithm is to document the collated data: Document the collated data on malaria disease, mosquito population, minimum temperature, maximum temperature, relative humidity 1, relative humidity 2, rainfall into MTM pattern cycles.

### Statistical analysis

Statistical software’s MINITAB ver. 14 was used for plotting graphs, performing correlation, linear and multiple regression analysis, partial autocorrelation, and calculations required for MTM algorithm [66].

## Results

Application of a wide array of statistical tools revealed that malaria transmission is solely dependent on climatic conditions and there is a strong relationship between climatic factors, mosquito population and malaria disease incidence in Visakhapatnam.

### Mapping of malarial disease in the district of Visakhapatnam

**Fig 3:**
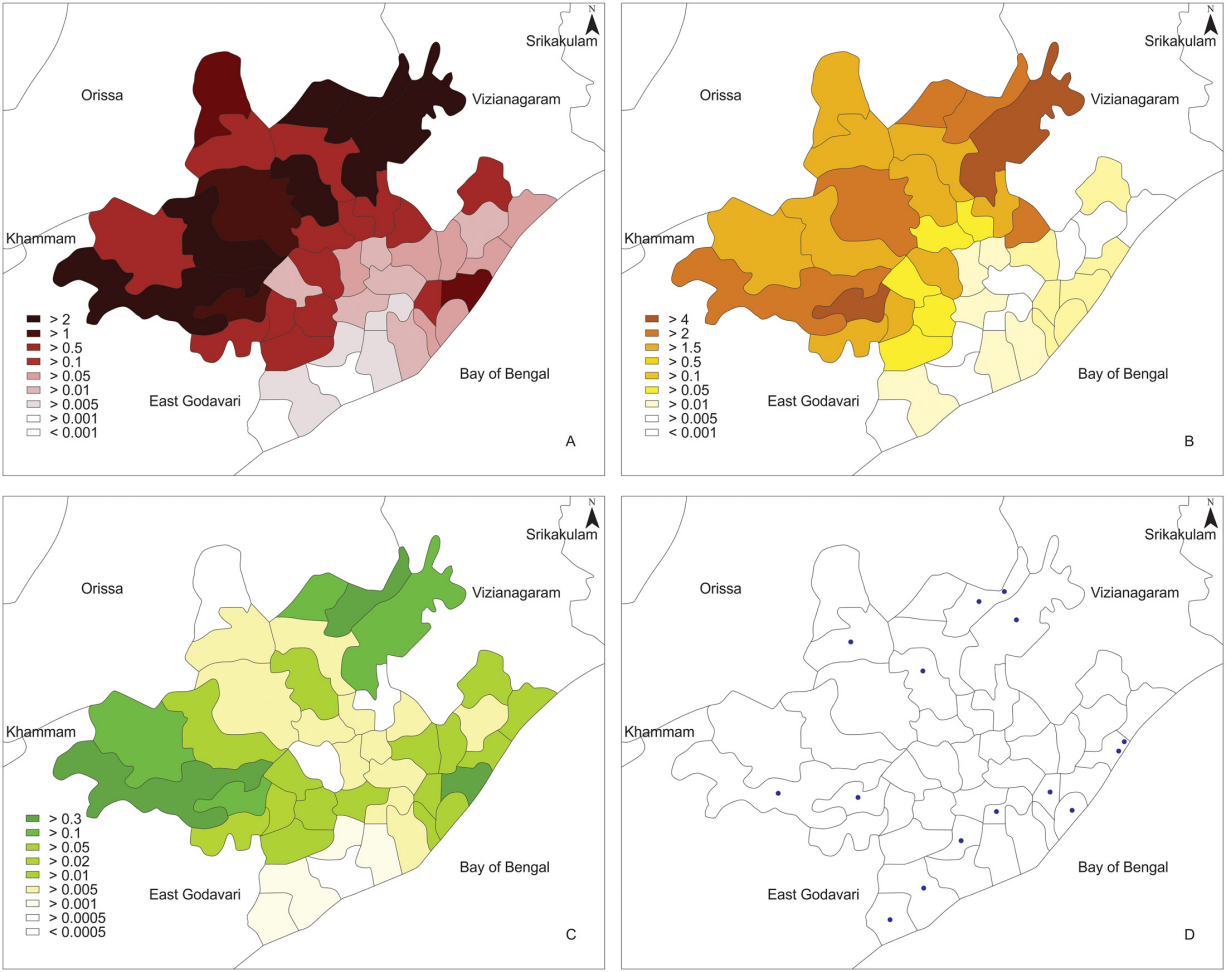
Maps developed for A) total malarial cases B) *P*. *falciparum* malarial cases C) *P*. *vivax* malarial cases and D) vector collection stations with reference to different mandals in District of Visakhapatnam.

Malaria disease density in 43 mandals of Visakhapatnam was mapped. Out of the 43 mandals in the district Paderu was having the highest rate of infection with 2%. Chintapalle, Koyyuru, Arakuvalley, Dumbriguda are having above 1% and the remaining mandals were below 1% of the infectivity rate (Fig 3A). Individual maps were also developed for *P*. *falciparum* (Fig 3B) and *P*. *vivax* (Fig 3C). In case of *P*. *falciparum*, Anantagiri was having the highest rate of infection with 4.2%. Golugonda, K.kotapadu, Koyyuru and the remaining mandals were showing 2%, 1.7%, and below 1% rate of infectivity respectively (Fig 3B). When compared with the total number of cases and *P*. *falciparum*, malaria disease incidence with *P*. *vivax* was low (Fig 3B), but it was observed that it still has an impact on the total population infectivity rate (Fig 3A).

Mosquito population data was available for 6 years from (2006–2011). The mosquito collection, field stations in Visakhapatnam are represented in Fig 3D. Mapping of the vector stations based on the data provides new insights. There is a need for new vector stations to be established in the mandals which are highly infected with malaria. This provides more appropriate information on the transmission cycle of malarial parasite.

### Statistical evaluation of climatic factors, mosquito populations and malarial disease incidence

**Fig 4:**
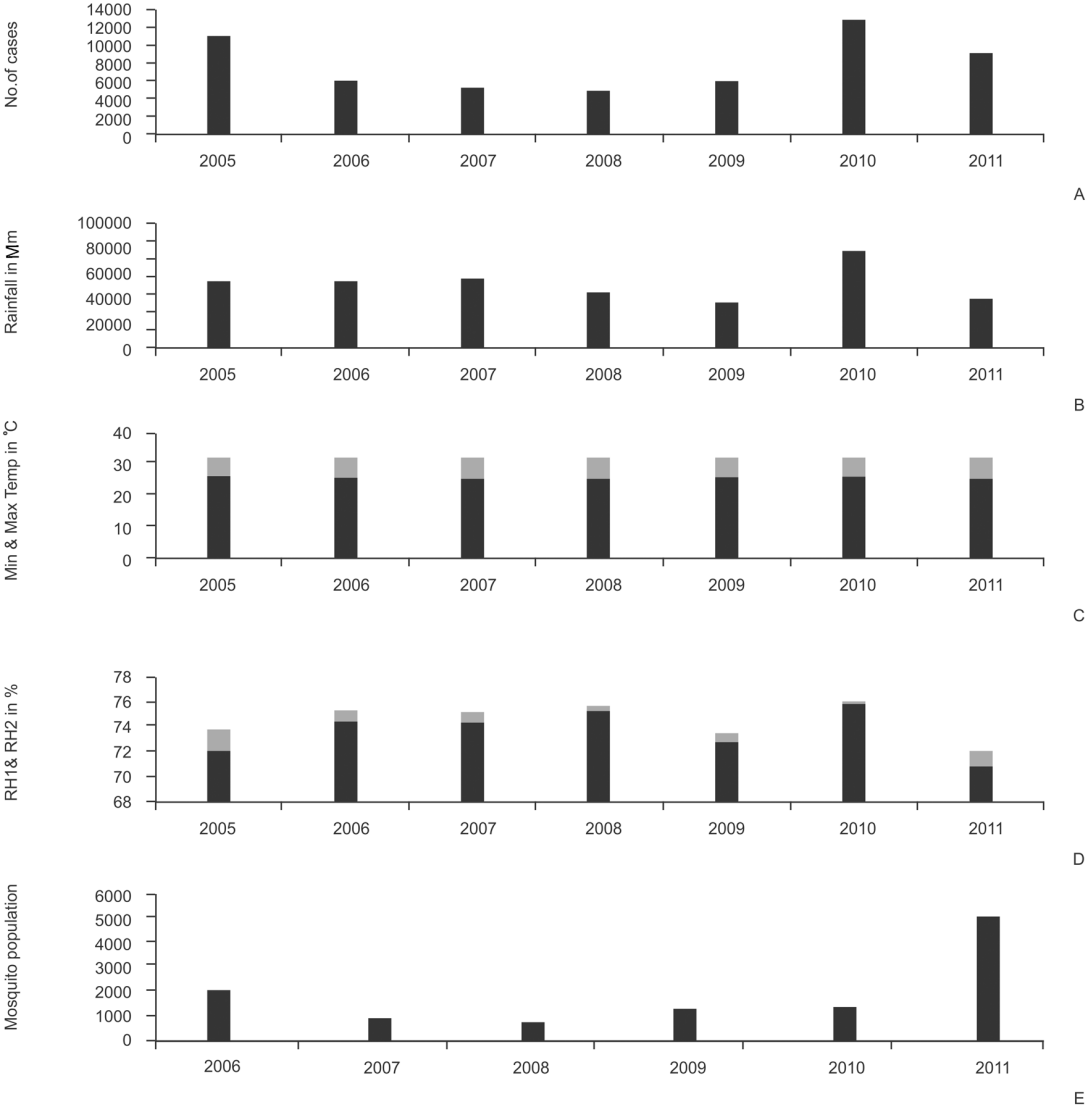
Annual data (yearly dataset) of a) malarial cases b) rainfall c) temperature d) humidity and e) mosquito populations in District of Visakhapatnam to understand the magnitude of the parameters.

Magnitude of malaria disease can be observed from the annual cases recorded. Annual cases recorded fluctuations of malaria disease incidence from the data for the period of 2005–2011 in the district of Visakhapatnam (Fig 4). An important trend was observed in this period, there was a rapid decrease in positive cases from the year 2005 and a slight increase in positive cases from the year 2010 (Fig 4). A similar trend was also observed in all other factors and parameters such as mosquito population data, rainfall, maximum and minimum temperature, relative humidity (Fig 4). During the years the maximum temperature, minimum temperature, relative humidity 1, relative humidity 2 and rainfall increased by 0.22°C, 0.63°C, 3.3%, 2.59% and 15822.4 mm respectively in the district.

**Fig 5:**
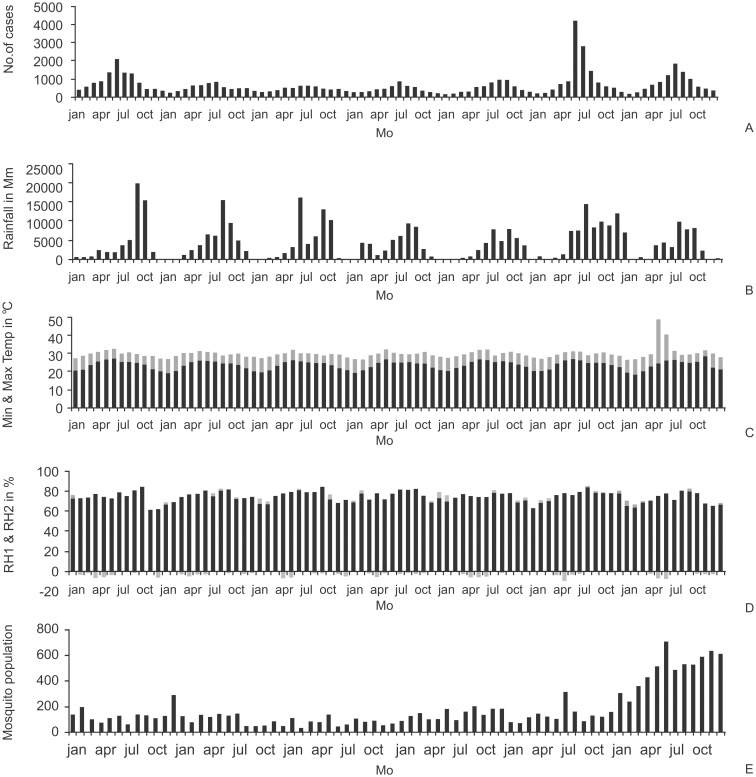
Monthly data (monthly dataset) for the years 2005–2011 on A) malarial cases B) rainfall C) temperature D) humidity and E) mosquito populations in District of Visakhapatnam to understand the trends of the parameters.

Trends of malarial disease were clearly evident from the Fig 5A for the years 2005–2011. Perennial malaria disease incidence was recorded in the district of Visakhapatnam. The highest and lowest for malarial disease, rainfall, average maximum and minimum temperatures, RH1 and RH2, and mosquito populations were as observed in Table 3 and Fig 5.

**Table 3:** Trends observed for climatic variables, mosquito population and malarial disease.

Year	Parameters	Rainfall	Minnimum Temperature	Maximum Temperature	Relative Humidity [Rh1] [8:00hrs]	Relative Humidity [Rh 2] [14:00hrs]	Mosquito Population	Disease cases
**2005**	**Highest**	September	June	May	October	October	-	June
	**Lowest**	December	December	December	December	November	-	December
**2006**	**Highest**	August	June	May	August	September	December	July
	**Lowest**	January	January	January	January	January	September	January
**2007**	**Highest**	September	May	May	September	September	July	July
	**Lowest**	January	January	January	November	February	February	January
**2008**	**Highest**	August	August	May	February	September	May	July
	**Lowest**	December	January	February	November	November	January	January
**2009**	**Highest**	September	June	May	July	July	September	July
	**Lowest**	January	January	January	December	December	January	January
**2010**	**Highest**	July	May	May	July	July	July	June
	**Lowest**	February	January	December	April	December	February	January
**2011**	**Highest**	July	October	May	August	July	June	July
	**Lowest**	January	January	January	November	January	February	January

#### Correlation of climatic factors, mosquito populations and malarial disease incidence

Positive significant relationship (r = 0.3440, p- value = 0.001) was observed between total monthly rainfall (mm) with the monthly malaria disease incidence for a period of seven years (2005–2011). The positive correlation coefficient value explains that rainfall has an impact on disease incidence in Visakhapatnam district, which is significant at the 5% level. Further, the highest significant relationship was observed between these two variables in the year 2009 (r = 0.908 and p value = 0.000). Whereas a positive, non-significant relationship was observed between total rainfall and mosquito population (r = 0.002, p = 0.988).

Average maximum temperature has a significant impact on disease (r = 0.3306, p = 0.002) at the 5% level for a period of seven years (2005–2011). The correlation between minimum temperature and malaria disease prevalence is highly significant (r = 0.4586 p = 0.0). The same is depicted in each and every year. Furthermore, the correlation between mosquito population and maximum temperature (r = 0.328, p = 0.007) shows a positive significant relationship, whereas a positive, non-significant relationship (r = 0.120, p = 0.334) with minimum temperature was observed at the 5% level (Table 4).

**Table 4:** Correlation coefficients between the climatic variables, mosquito population and malarial disease.

Year	Parameters	Rainfall	Minimum Temperature	Maximum Temperature	Relative Humidity [Rh1] [8:00hrs]	Relative Humidity [Rh 2] [14:00hrs]	Mosquito Population	Disease cases
**2005**	**Disease cases**	0.1053	0.8480	0.8933	0.1382	0.2542	-	-
	**Mosquito Population**	-	-	-	-	-	-	-
**2006**	**Disease cases**	0.3944	0.8714	0.7952	0.5634	0.5848	0.5339	-
	**Mosquito Population**	-0.2831	-0.6549	-0.5825	-0.2032	-0.1839	-	0.5339
**2007**	**Disease cases**	0.5095	0.9014	0.7594	0.6244	0.7785	0.8172	-
	**Mosquito Population**	-0.1862	0.2821	0.3806	0.1047	0.1275	-	0.8172
**2008**	**Disease cases**	0.7033	0.7148	0.4913	0.4112	0.7143	0.7888	-
	**Mosquito Population**	-0.0072	0.2863	0.4237	-0.3884	-0.1976	-	0.7888
**2009**	**Disease cases**	0.9075	0.6606	0.5206	0.4572	0.5569	0.7759	-
	**Mosquito Population**	0.2399	-0.0013	0.1258	-0.1906	-0.1278	-	0.7759
**2010**	**Disease cases**	0.4403	0.5121	0.4880	0.5032	0.5926	0.7401	-
	**Mosquito Population**	0.5777	0.2051	-0.0149	0.5324	0.4782	-	0.7401
**2011**	**Disease cases**	0.9003	0.6079	0.1835	0.8385	0.8201	0.8371	-
	**Mosquito Population**	0.2045	0.5796	0.2948	0.0235	0.0860	-	0.8371

Significant association was revealed between relative humidity values (RH 1 –r = 0.3029, p = 0.005; RH 2 –r = 0.3888, p = 0.00) and malaria cases, demonstrating the realistic situation of the association between malaria cases and relative humidity. Whereas a negative insignificant correlation was observed between the mosquito population with relative humidity 1 (r = -0.158, p = 0.201) and relative humidity 2 (r = -0.058, p = 0.640) at the 5% level (Table 4). A positive significant correlation was also observed between monthly total mosquito population and monthly malaria disease incidence from 2006 to 2011 (r = 0.257 p = 0.036).

#### Seasonal patterns and index

**Fig 6:**
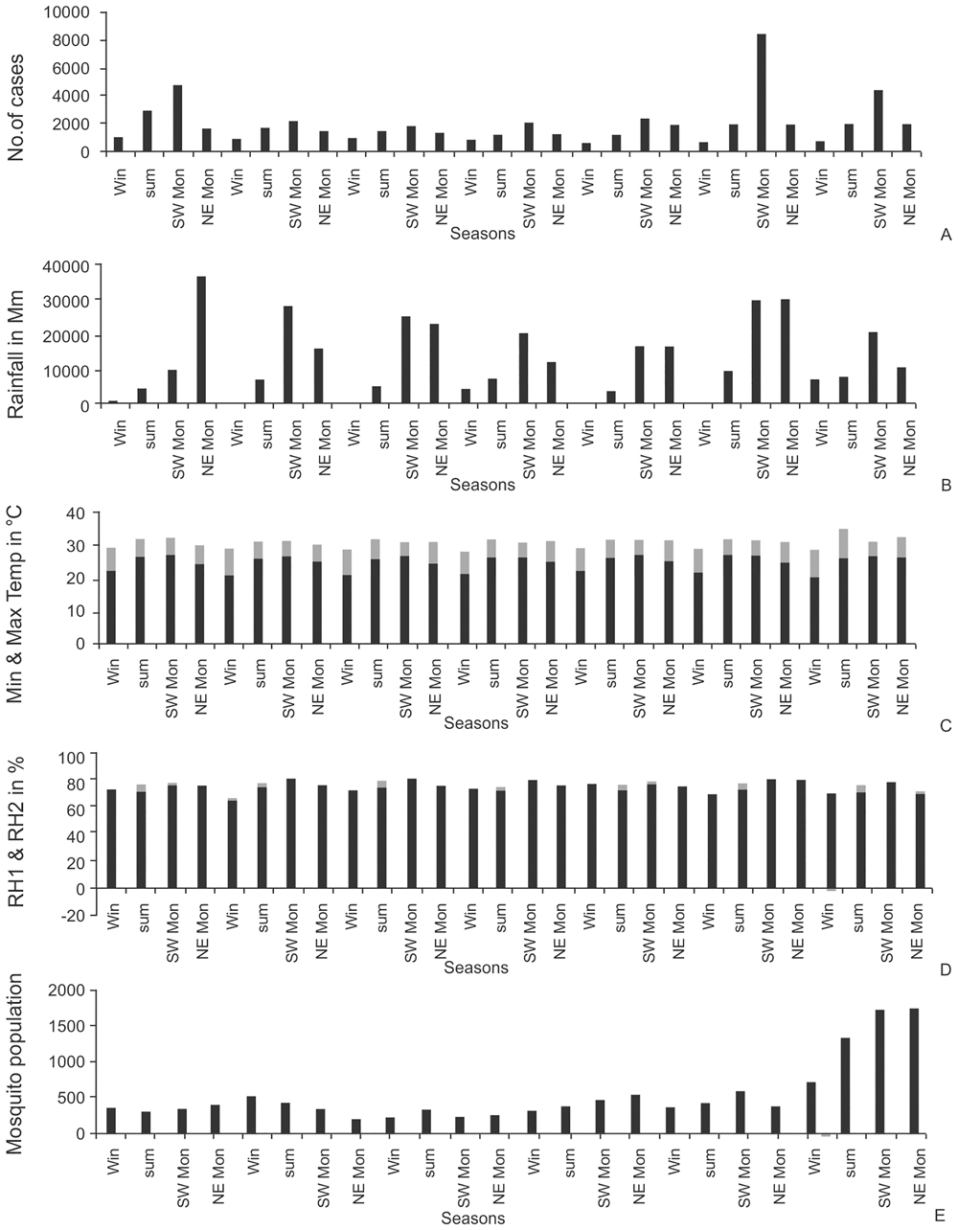
Seasonal data (quarterly dataset) of A) malarial cases B) rainfall C) temperature D) humidity and E) mosquito populations in District of Visakhapatnam to understand the effect of seasons on malaria disease.

The seasonal patterns of the malaria disease incidence, climatic factors and mosquito population are shown in Fig 6. Changes in seasonal climatic conditions are mostly responsible for the fluctuations in the disease incidences in each and every month of the year (Table 5). T-test between two methods of seasonal indexes for all the variables using the original values demonstrated that there was no significant variation (T-value - 0.01, P-value - 0.989). Though there was no significant variation between two methods, it is best to implement the new proposed method than the Barnett and Dobson, [62] as the new method takes in seasons of the study area into consideration for the calculation of seasonal index. Statistically significant variation of monthly malarial cases (p = 0.036) (Fig 6) was observed in the study area in almost every month of the year. The highest peak of malarial cases in almost all years was observed during September with an exception in 2009, in this case the highest number malarial cases were observed during October. Seasonal index has shown that maximum number of cases were occurring in the third quarter. Most of the cases in the third quarter were increased after the second quarter. Minimum numbers of cases were recorded in the first and fourth quarter. So, the probable trend that can be observed is that the numbers of cases are increasing from the second quarter to third quarter, followed by a decrease in the number of cases in the fourth quarter and subsequently a decrease in the number of cases in the first quarter.

**Table 5:** Seasonal index of malaria cases during the years 2005–2011 third and second quarters has the highest incidence were as lowest occurred in first and fourth quarters.

Parameters	Barnett and Dobson, 2010				Proposed Method			
	Quarters				Quarters			
	Q1	Q2	Q3	Q4	Win	Sum	Mon1	Mon2
**Malarial Cases**	0.61	1.31	1.40	0.68	0.39	0.89	1.90	0.82
**Rainfall**	0.16	0.92	2.05	0.87	0.16	0.51	1.70	1.63
**Minimum Temperature**	0.91	1.11	1.01	0.96	0.86	1.06	1.08	1.01
**Maximum Temperature**	0.95	1.07	1.01	0.98	0.93	1.05	1.02	1.00
**Relative Humidity 1**	0.98	0.98	1.08	0.96	0.96	0.97	1.05	1.01
**Relative Humidity 2**	0.96	1.03	1.07	0.95	0.93	1.01	1.05	1.00
**Mosquito Population**	0.78	1.04	1.04	1.13	0.75	0.98	1.17	1.09

### Modeling and prediction of climatic factors, mosquito populations and malarial disease incidence

Development of suitable statistical models to study and predict the association between climatic factors and the malaria disease incidence is most important in the application of control measures and implementation of best possible procedures. Modeling association of mosquito population with climatic factors gives us the information which factors are most responsible for their distribution and development.

#### Regression analysis

The relationship between malaria parasite prevalence and each individual potential explanatory variable was performed. Each of the explanatory variables was adjusted for all of the others by performing multiple regressions in the usual way.

#### Linear regression analysis

Linear regression analysis of monthly data revealed that total rainfall, monthly average maximum temperature, the average minimum temperature, relative humidity 1 and relative humidity 2 showed significant variation for malarial cases due to climatic factors at 5% level of significance (Table 6). At the same time from the R-square values and its corresponding P-values there is a significant variation in mosquito population due to climatic factor like monthly average maximum temperature and relative humidity 1, whereas the remaining factors total rainfall, monthly average minimum temperature, and relative humidity 2, does not have an impact on mosquito population at the 5% level of significance (Table 6). Linear regression analysis on seasonal data revealed that total rainfall, monthly average maximum temperature, average minimum temperature and relative humidity 2 showed significant variation for malarial cases at 5% level of significance, whereas relative humidity 1, average rainfall and mosquito population does not have an impact on malarial cases at 5% level of significance (Table 6). At the same time from the R-square values and its corresponding P-values, climatic factors do not have an impact on mosquito population at the 5% level of significance (Table 6).

**Table 6:** Linear regression method for malarial cases and mosquito population using malaria disease cases, rainfall, temperature, relative humidity and mosquito populations.

	**Monthly Data**					
	**Malaria Cases**			**Mosquito Population**		
	**R-Sq**	**P**	**Decision**	**R-Sq**	**P**	**Decision**
**Total Rainfall in mm**	11.8	0.001	Significant	0.1	0.751	Non Significant
**Minimum Temperature**	23.6	0.000	Significant	1.8	0.259	Non Significant
**Maximum Temperature**	10.9	0.002	Significant	6.8	0.027	Significant
**Relative Humidity 1**	9.2	0.005	Significant	5.6	0.046	Significant
**Relative Humidity 2**	15.1	0.000	Significant	3.0	0.143	Non Significant
	**Seasonal Data**					
	**Malaria Cases**			**Mosquito Population**		
	**R-Sq**	**P**	**Decision**	**R-Sq**	**P**	**Decision**
**Total Rainfall in mm**	23	0.0278	Significant	0.0015	0.9873	Non Significant
**Minimum Temperature**	45.39	0.00083	Significant	0.9	0.9025	Non Significant
**Maximum Temperature**	27.24	0.015	Significant	5.2	0.3475	Non Significant
**Relative Humidity 1**	8.2	0.208	Non Significant	1.43	0.6255	Non Significant
**Relative Humidity 2**	32.34	0.0071	Significant	4.37	0.3906	Non Significant

#### Multiple regression analysis

**Fig 7:**
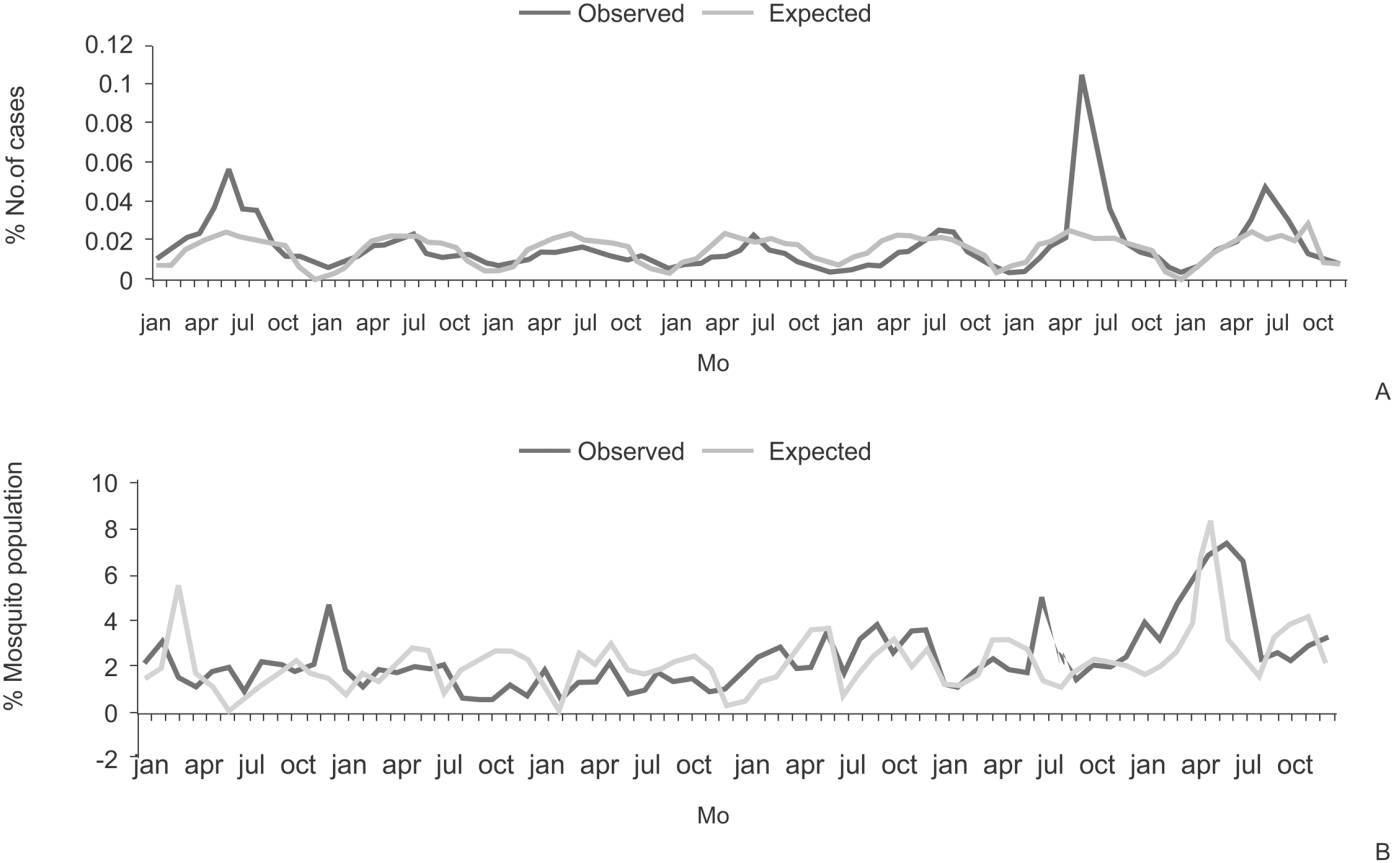
Graph on observed and expected numbers predicted using multiple regression method A) number of malarial cases and B) mosquito populations.

Multiple regression analysis of monthly data illustrates that 27% variation was established due to the independent variables such as total rainfall, average rainfall, minimum temperature, maximum temperature, relative humidity 1 and relative humidity 2. P-values suggest that, except the independent variable minimum temperature the remaining variables do not have a significant impact on the percentage (%) number of cases (Table 7). Further, the expected percentage number of cases in the 7 years (2005–2011) was calculated month wise using multiple regression analysis. Chi-square analysis test proposes that there is no significant difference between the observed percentage number of cases and expected percentage number of cases with a chi-square value (0.9326) which is significant (P-value = 1.000) and suggests that the given model is best fit to the given data (Fig 7).

**Table 7:** Multiple regression method to model malarial cases and mosquito population using malaria disease cases, rainfall, temperature, relative humidity and mosquito populations.

	**Monthly data**							
	**Model on Malarial Cases**				**Model on Mosquito Population**			
	**Coefficient of variable**	**Standard Error of Coefficient**	**Test value**	**P-value**	**Coefficient of variable**	**Standard Error of Coefficient**	**Test value**	**P-value**
**Constant**	-0.06477	0.04067	-1.59	0.115	473.7	561.3	0.84	0.402
**Total Rainfall in mm**	0.00000043	0.00000056	0.77	0.446	0.009554	0.007688	1.24	0.218
**Avgerage Rainfall**	-0.00000238	0.00001156	-0.21	0.837	-0.0632	0.1285	-0.49	0.625
**Minimum Temperature**	0.002914	0.001243	2.34	0.022	10.00	14.65	0.68	0.497
**Maximum Temperature**	-0.000043	0.001305	-0.03	0.974	21.28	14.87	1.43	0.157
**Relative Humidity 1**	0.0004318	0.0006663	0.65	0.519	-3.232	9.100	-0.36	0.724
**Relative Humidity 2**	-0.0002962	0.0007236	-0.41	0.683	-13.416	8.791	-1.53	0.132
	R-Sq = 27.0%, P-value: 0.000				R-Sq = 18.3%, P-value: 0.035			
	**Seasonal data**							
	**Model on Malarial Cases**				**Model on Mosquito Population**			
	**Coefficient of variable**	**Standard Error of Coefficient**	**Test value**	**P-value**	**Coefficient of variable**	**Standard Error of Coefficient**	**Test value**	**P-value**
**Constant**	1691	3481	0.49	0.637	510	1558	0.33	0.749
**Total Rainfall in mm**	0.04888	0.05168	0.95	0.365	0.01995	0.02252	0.89	0.393
**Avgerage Rainfall**	-0.348	1.124	-0.31	0.762	-0.0613	0.5051	-0.12	0.905
**Minimum Temperature**	100.8	129.3	0.78	0.452	54.37	55.97	0.97	0.351
**Maximum Temperature**	-167.4	133.5	-1.25	0.236	70.77	56.45	1.25	0.234
**Relative Humidity 1**	-69.19	64.94	-1.07	0.310	24.68	28.32	0.87	0.401
**Relative Humidity 2**	85.90	89.30	0.96	0.357	-77.54	33.34	-2.33	0.038
	R-Sq = 60.8%, p- value = 0.09				R-Sq = 55.3%, p- value = 0.086			

Multiple regression analysis of mosquito population data illustrates that 18.3% variation was established due to the independent variables like monthly total rainfall, monthly average minimum and maximum temperatures and average relative humidity 1 and 2 (Table 7). Further, the expected percentage number of mosquito population in the 6 years (2006–2011) was calculated month wise using the multiple regression model and was subjected to chi-square analysis. Chi-square test (34.85) administered with insignificant p-value (0.05), illustrates the model was best fit to the data (Fig 7). Multiple regression analysis of seasonal data for malaria disease and mosquito population was not significant.

#### Autoregressive analysis

**Fig 8:**
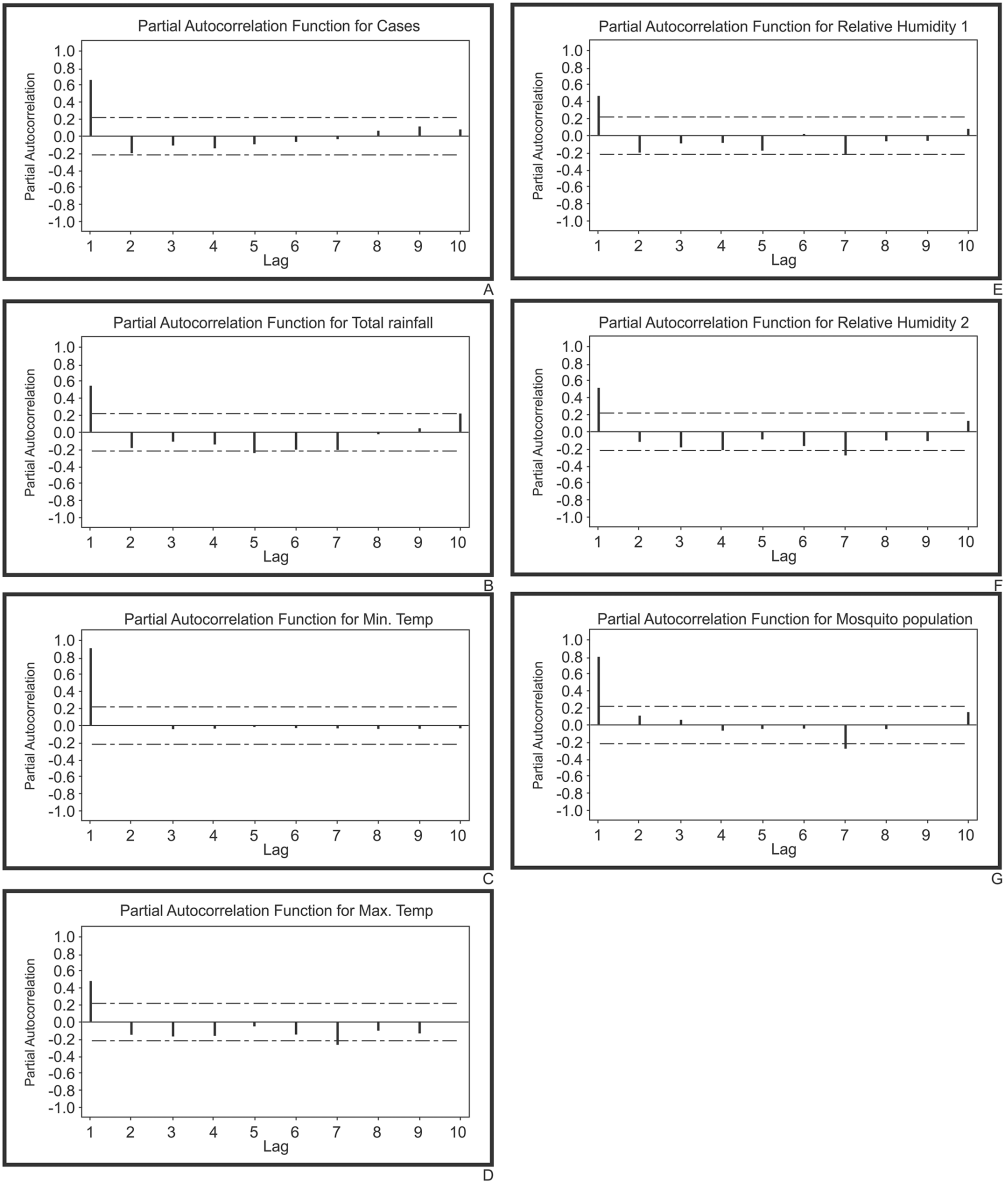
Partial autocorrelations coefficients on monthly data for A) malarial cases B) total rainfall C) minimum temperature D) maximum temperature E) relative humidity 1 F) relative humidity 2 and G) mosquito populations in District of Visakhapatnam to understand the order of ARIMA (1) model.

**Fig 9:**
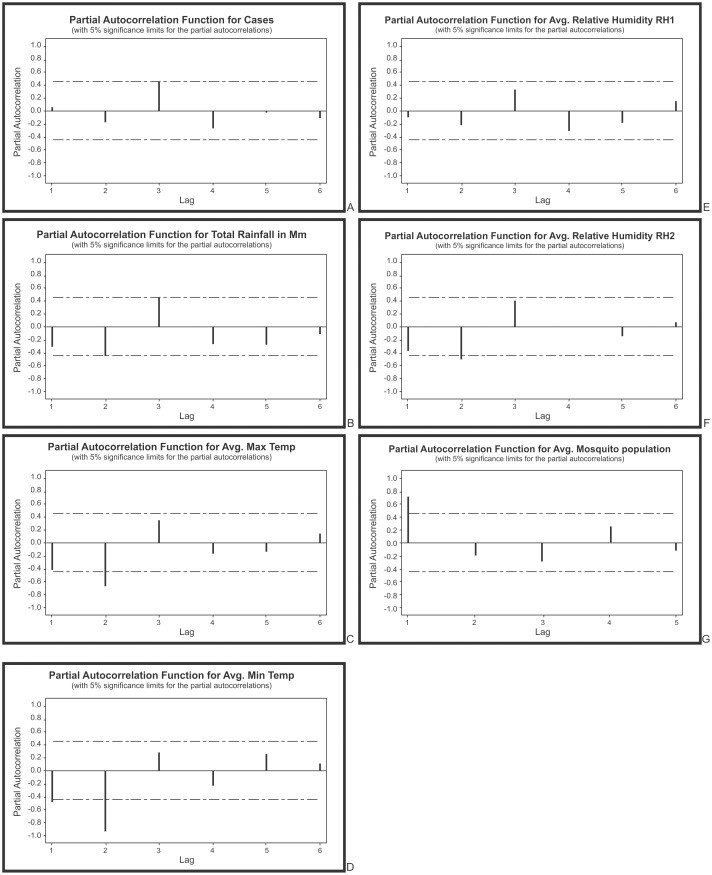
Partial autocorrelations coefficients on seasonal data for A) malarial cases B) total rainfall C) minimum temperature D) maximum temperature E) relative humidity 1 F) relative humidity 2 and G) mosquito populations in District of Visakhapatnam to understand the order of ARIMA (1) model.

Autoregressive analysis of monthly data concluded that, the partial autocorrelation functions for all the seven come under the ARIMA model order one, i.e., there is only single large spike at lag 1 for the montly data, which is typical of an autoregressive process of order one with the equation Y = a + bx. Further, from seasonal data insignificant spikes at the mentioned lags for the variables total rainfall (5^th^ lag), minimum temperature (3^rd^ lag), maximum temperature (7^th^ lag), relative humidity 1 (7^th^ lag), relative humidity 2 (7^th^ lag) and mosquito population (7^th^ lag) concluding no evidence of non- randomness process (Fig 8). Autoregressive analysis of seasonal data for malaria disease and mosquito population was not significant (Fig 9).

Based on the above mentioned multiple regression and ARIMA (I) models predictions were made upto 2030 for parameters like malarial disease cases, total rainfall, minimum temperature, maximum temperature, relative humidity and mosquito population (Table 8). Predictions on variables like disease cases, minimum temperature, maximum temperature and mosquito population were increasing by the year, whereas total rainfall, relative humidity1, and relative humidity 2 were decreasing with the year.

**Table 8:** Forecasted values modeled using ARIMA model and multiple regressions for malarial disease cases, rainfall, minimum temperature, maximum temperature, relative humidity’s and mosquito populations.

Year	Cases	Total rainfall	Average Rainfall	Minimum Temperature	Maximum Temperature	Relative Humidity 1	Relative Humidity 2	Mosquito Population
	**ARIMA model**							
**2013**	6170	3098.14	113.58	23.23	30.68	72.32	72.63	5653
**2015**	7126	3962.91	115.58	23.86	30.98	73.25	74.11	4462
**2020**	7761	4315.72	115.62	24.52	31.07	73.50	74.58	2809
**2025**	7383	4333.16	115.62	24.68	31.07	73.50	74.60	2121
**2030**	7848	4334.02	115.62	24.73	31.07	73.50	74.60	1836
	**Multiple Regression Method**							
**2013**	9403	3297	74.83	24.66	30.35	73.19	73.66	5141
**2015**	10085	3257	58.65	24.80	30.56	73.07	73.30	6237
**2020**	11790	3157	18.2	25.13	31.08	72.76	72.40	8977
**2025**	13495	3057	-22.25	25.47	31.60	72.45	71.50	11717
**2030**	15200	2957	-62.7	25.80	31.12	72.14	70.60	14457

### MTM patterns identified using MTM algorithm

Climatic conditions in Visakhapatnam are suitable for transmission of malaria disease. Malarial transmission mechanism cycle starts with increase in mosquito population from the available initial mosquito population. The observed data (2006–2011) showed that initial mosquito population ranged from 30 to 550 nos. For the period 2006–2011, climatic factors like rainfall (5–1311%), minimum temperature (1.6–15.7%), maximum temperature (0.5–33.8%), relative humidity 1 (0.1–16.6%) and relative humidity 2 (0.9–13.8%) demonstrated fluctuations in the data. Anomalies observed in the above data in the district of Visakhapatnam can be attributed to the fluctuations in climatic factor that favored an increase in mosquito populations and thereby increase in number of malarial cases. Following fluctuations in climatic factors an increase in mosquito population (6.7–170%) was found in the same/next month (Table 9). Fluctuations in the climatic conditions were observed between the months of February to September the data (2005–2006). In the months of March and June of every year definite fluctuations in the climatic conditions were observed from the data (Table 9).

**Table 9:** Fluctuations in rainfall, temperature and relative humidity observed for the period 2006–2009 forming the basis for increase in mosquito populations and malarial cases.

Years	Months	Rainfall	Min.Temp	Max.Temp	RH 1	RH 2	M.P	Cases
**2006**	**February**	0.0↑	5.5↑	5.8↑	1.3↑	4.2↑	7.1↑	-29.2↓
	**March**	1317.0↑	15.2↑	5.0↑	4.3↑	7.1↑	42.2↑	33.6↑
	**April**	85.3↑	8.2↑	0.3↑	1.3↑	3.6↑	-49.0↓	39.3↑
	**May**	53.3↑	2.7↑	3.2↑	2.7↑	0.4↑	-27.6↓	47.2↑
	**June**	72.6↑	-0.1↓	-1.5↓	4.8↑	4.4↑	52.1↑	5.6↑
	**July**	-5.9↓	-2.4↓	-0.9↓	-0.1↓	-6.7↓	15.7↑	18.3↑
	**August**	152.5↑	-3.8↓	-4.8↓	5.4↑	8.0↑	-53.6↓	7.6↑
**2007**	**February**	267.7↑	5.4↑	3.5↑	3.5↑	-4.9↓	-37.2↓	15.4↑
	**March**	117.4↑	13.5↑	4.6↑	4.4↑	8.5↑	71.1↑	24.0↑
	**April**	179.8↑	7.8↑	2.3↑	2.3↑	-4.5↓	-8.5↓	32.3↑
	**May**	94.5↑	4.2↑	6.0↑	6.3↑	2.5↑	18.5↑	-0.8↓
	**June**	400.7↑	-1.1↓	-5.4↓	-5.4↓	11.0↑	-9.2↓	21.9↑
	**July**	-74.6↓	-3.0↓	0.6↑	-0.6↓	-3.2↓	11.7↑	1.5↑
**2008**	**February**	3660.9↑	7.9↑	-0.7↓	14.6↑	12.4↑	-72.0↓	6.8↑
	**March**	-5.6↓	7.8↑	8.9↑	-13.2↓	-8.0↓	170.0↑	18.3↑
	**April**	-73.9↓	9.8↑	3.3↑	3.4↑	8.4↑	-7.4↓	37.6↑
	**May**	130.6↑	7.7↑	7.3↑	-2.5↓	-7.1↓	78.7↑	7.3↑
	**June**	111.4↑	-5.4↓	-6.2↓	8.4↑	8.5↑	-67.9↓	29.3↑
	**July**	19.0↑	-2.7↓	-0.5↓	-3.1↓	-1.9↓	30.2↑	48.7↑
	**August**	53.1↑	-2.1↓	-0.8↓	-1.6↓	0.2↑	87.5↑	-30.9↓
**2009**	**February**	0.0↑	10.4↑	4.2↑	-2.9↓	5.4↑	40.9↑	21.9↑
	**March**	419.8↑	4.5↑	3.0↑	2.1↑	4.5↑	17.7↑	52.2↑
	**April**	62.8↑	10.7↑	4.5↑	-6.7↓	-2.0↓	-31.5↓	1.1↑
	**May**	264.7↑	3.6↑	2.3↑	-1.1↓	-1.5↓	-2.0↓	96.0↑
	**June**	68.9↑	0.0↑	0.0↑	0.5↑	0.0↑	81.6↑	7.8↑
	**July**	85.6↑	-6.0↓	-9.5↓	16.6↑	6.5↑	-50.0↓	36.8↑
	**August**	-37.4↓	3.0↑	3.9↑	-5.4↓	-1.8↓	80.9↑	17.7↑
	**September**	61.1↑	-1.9↓	2.1↑	1.7↑	0.9↑	24.2↑	0.1↑
**2010**	**February**	-92.3↓	4.8↑	3.5↑	2.8↑	2.6↑	-9.2↓	13.1↑
	**March**	599.6↑	15.7↑	4.5↑	0.8↑	8.5↑	66.7↑	112.6↑
	**April**	247.5↑	6.7↑	6.4↑	-6.0↓	2.4↑	24.3↑	68.6↑
	**May**	461.5↑	3.2↑	2.4↑	6.0↑	-2.4↓	-19.6↓	23.5↑
	**June**	-0.3↓	-3.3↓	-0.2↓	4.9↑	4.0↑	-11.3↓	401.3↑
	**July**	93.7↑	-4.6↓	-7.3↓	9.6↑	5.3↑	205.9↑	-33.6↓
	**August**	-42.6↓	-49.3↓	2.8↑	-5.2↓	-6.1↓	-50.0↓	-49.3↓
	**September**	18.2↑	-45.5↓	1.5↑	-1.5↓	-0.5↓	-48.1↓	-45.5↓
**2011**	**February**	460.4↑	10.2↑	5.8↑	5.5↑	8.5↑	-20.8↓	43.0↑
	**March**	-75.1↓	13.2↑	4.5↑	-1.6↓	2.7↑	49.6↑	85.6↑
	**April**	3096.3↑	7.8↑	2.7↑	0.1↑	6.6↑	19.5↑	50.9↑
	**May**	17.9↑	5.6↑	33.3↑	2.6↑	3.4↑	20.0↑	21.3↑
	**June**	-27.4↓	1.7↑	-22.1↓	0.7↑	-8.9↓	6.8↑	48.8↑
	**July**	211.5↑	-5.0↓	-7.1↓	12.8↑	13.9↑	-11.5↓	53.1↑

## Discussion

The development of malaria early warning systems [67, 68] to predict malarial epidemics for prevention activities was in need [56, 63, 70–72]. However, little consensus has emerged as to which factors can be used as malarial indicators, because multiple studies have yielded different results [22, 73–77].

### Malarial disease maps of Visakhapatnam

Malaria parasite transmission intensity is spatially heterogeneous [63, 67–69] and this heterogeneity has important implications [69, 70]. Good maps of malaria risk have long been recognized as an important tool for malaria control. The production of such maps is useful to predict the risk of progression of malaria transmission mechanism in the mandals of Visakhapatnam district. Mapping of the total number of malaria cases in the district of Visakhapatnam resulted in identifying the mandals with high rate of infection with the malarial parasite for targeted control of disease (Fig 3).

### Statistical evaluation of climatic factors, mosquito populations and malarial disease incidence

Many studies on epidemics and time-series have worked out to find explanatory variables for changes in malaria transmission, but many of them failed to take climate factors into an account. Factors other than climate that affect malaria are urbanization, migration, irrigation, agricultural practices, deforestation and malaria control efforts.

Analysis carried out at monthly time scale, established a strong temporal link between climatic indices and increasing risk for malaria disease. In China [24], India [33, 78] and Sudan [79], monthly malaria incidences and transmission of the disease were positively correlated with monthly mean climatic variables (relative humidity, temperature and rainfall). Analysis established a strong link between climatic indices and mosquito populations similar results were observed with Grace [80], Beck-Johnson et al., [81], Blanford et al., [82], and Patz and Olson [83].

Analysis in the study area identified monthly total rainfall as the significant factor for malaria transmission. It was revealed that the increase in rainfall plays an important role in malaria epidemiology. Life cycle of mosquitoes is dependent on water, rainfall provides the medium for breeding of mosquitoes to lay their eggs and their development is indirectly contributed by suitable relative humidity (50 to 60%) which is optimal for survival of *Anopheles* mosquito to acquire and transmit the parasite. If rainfall is moderate it is beneficial for mosquito breeding, but it is excessive it may destroy breeding sites and flush out the mosquito larvae [84]. Apart from rainfall, some other non-climatic factors, such as road and other construction activities increased the number of breeding sites of mosquitoes in the study area (personal observation). In some Sub-Saharan countries, malaria transmission is restricted to the rainy seasons [85], the same situation is observed in the district of Visakhapatnam. However, the effect of rainfall on the malaria transmission is complicated and varies with local habits of mosquitoes and geographical regions.

Analysis identified monthly temperature as most significant factor in the study area. Temperature with range of 16°C to 36°C are considered suitable for the survival and development of mosquito [86]. Analysis in the study area identified monthly maximum temperature as the significant factor for malaria disease and mosquito population. Temperature above 36°C compromise development and survival of the mosquito. The rise in maximum temperature during the seven years has increased up to 0.2°C and disease cases also slightly increased in 2009, 2010 and 2011. Pletsch [87] reported biological amplification of temperature effects and stated that an increase of 0.5°C in temperature can show a trend with an increase mosquito abundance by 30–100%. The gradual increase in the maximum temperature from 28°C to 32°C January to April, followed by gradual decrease from July to December can be correlated to the gradual increase of malarial cases from January to June followed by gradual decrease from July to December. Therefore, maximum temperature between 28°C to 30°C in the district of Visakhapatnam are favoring survival of malaria vectors and thereby transmission of malarial parasite to host.

The development rate of *P*. *falciparum* and *P*. *vivax* is temperature dependent. Minimum temperature is required for development of parasite *P*. *falciparum* and *P*. *vivax* [87]. Temperatures with range of 16°C to 36°C are considered suitable for *Plasmodium* sps development and transmission through the mosquito. Analysis in the study area identified monthly minimum temperature as the next most significant factor for malarial disease. Minimum temperature range of 20°C to 28°C (S3 Dataset) also strongly influences the transmission of malaria in the district of Visakhapatnam. Temperature below 16°C ceases the development of the parasite, whereas according to the Paaijmans curve parasite development will above 30°C and eventually halt at 35°C [81]. According to The United Nations Intergovernmental Panel on Climate Change (IPCC) (2007) [88] rise in minimum temperature may allow mosquito-borne diseases to spread into regions free of disease. The gradual increase in the minimum temperatures from 20 to 28°C from January to June, followed by gradual decrease from July to December can be correlated to the increase of malarial cases from January to June followed by gradual decrease from July to December. Therefore, minimum temperature between 20°C to 33°C in the district of Visakhapatnam are favoring transmission of malaria.

**Fig 10:**
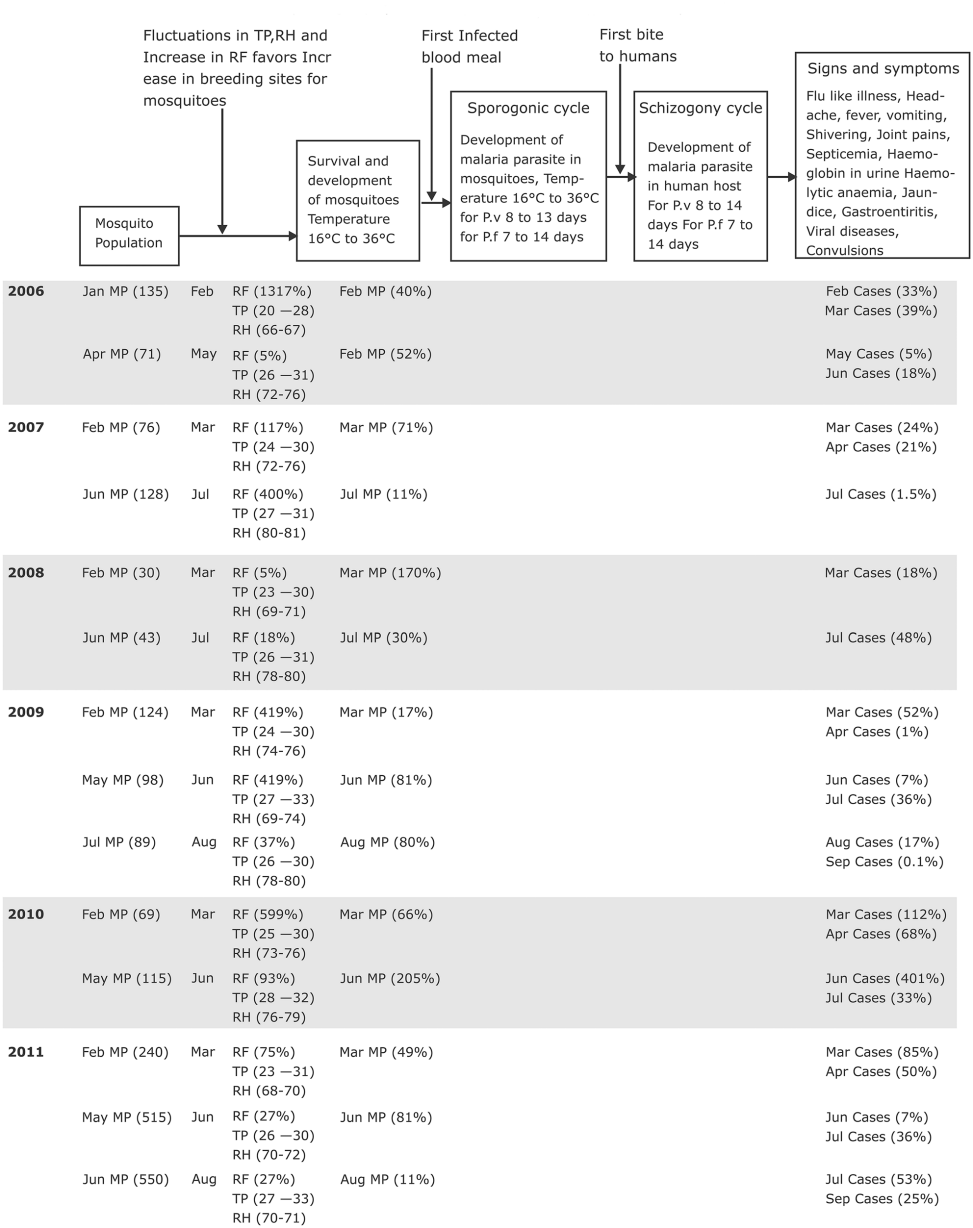
MTM pattern cycles observed in district of Visakhapatnam during year 2006–2011. Climatic factors influence mosquito populations thereby effecting MTM.

Relative humidity is always higher and greater than 60% in Visakhapatnam. Humidity is also considered as a requirement for the mosquito to survive long enough for the parasite to develop sufficiently and then transmitted to its host human. So, rainfall for mosquitoes, temperature between 20°C to 33°C and humidity with a range of 66% to 81% in the district of Visakhapatnam maintained a warmer, wetter climate that will lead to a longer period for mosquito growth, parasite development with higher potential for malaria transmission (Fig 10).

The season with the highest average total malaria cases occurrence was spring (June, July and August) and the minimum malaria cases were observed during the winter (December, January and February). For total malaria cases, the seasonal variation was statistically significant (p = 0.007). At species level, both *P*. *vivax* and *P*. *falciparum* maximum cases were observed in spring followed by autumn (March, April and May) and the minimum being during the winter followed by summer (June, July and August). In all seasons *P*. *falciparum* cases were higher than *P*. *vivax*. In years of high malaria cases, the spring peak was more pronounced when compared with other years and there was a substantial number of cases late in the year. The seasonal index for disease incidences is higher in tribal areas than in urban and rural areas, changes in climatic conditions is most responsible for the fluctuations in disease incidences in Visakhapatnam district. Seasonal index for rainfall is higher in rural areas than in urban and tribal areas.

Non-significant nature of the seasonal modeling can be attributed to the perennial existence of malarial cases in Visakhapatnam, with more prevalence in rainy seasons. These findings suggest that rigorous monitoring and preventive measures are required all round the year.

### Modeling and Prediction of climatic factors, mosquito populations and malarial disease incidence

Statistical modeling implies a formalization of relationships between two variables in the form of mathematical equations. A model describes one or more dependent or independent variables that are related to one or more variables. The models are applied to estimate parameters, assess numerical outputs, and predict future values from past observations. The use of statistical methods is increasing day by day for modeling and prediction of infectious diseases. Individual-based detailed modeling is used to study infectious disease epidemiology [89, 90]. Models predicted an increase in malaria disease by 10.98%, 23.85%, 33.47%, 40.94% for the following years 2015, 2020, 2025, 2030 respectively, whereas for mosquito populations an increase of 4.70%, 34.02%, 49.44%, 59.03% was predicted for the years 2015, 2020, 2025, 2030 respectively.

### MTM patterns in the district of Visakhapatnam

Analyzing all the dependent and independent variables from year 2006 to 2011 shows us that there is existence of MTM pattern cycles. Malarial transmission cycle starts with survival and development of mosquitoes due to fluctuations of climatic factors, followed by infection of blood meal by mosquitoes. After, ingestion of the first infected blood meal the malaria parasite develops (sporogonic cycle) in the gut of the female anopheles mosquito and transmits the disease in humans through biting. Then the malarial parasite develops (schizogony cycle) and display the following signs and symptoms in the host human like headache, fever, vomiting, shivering, joint pains, septicemia, haemoglobin in urine, haemolytic anaemia, jaundice, gastroenteritis, viral diseases, convulsions etc.

The time observed to initiate and complete the above described cycle of malaria parasites is MTM cycle. Generally, the previous reports characterized that major MTM cycles followed the June to September rains and occurred between September to December while the minor MTM cycles occur between April to May following the February to March rains. Whereas our observation demonstrates that a major MTM cycle followed the June to August rains and occurred between June to September and minor MTM cycles followed March to April rains and occurred between March to April (Fig 10).

We report two MTM cycle pattern and three MTM cycle pattern per year. In the year 2006, 2007, 2008 and 2010 two MTM cycle pattern was observed, whereas in the year 2009 and 2011 we observed three MTM cycle pattern. The time duration for the completion one transmission cycle is 26 days to 46 days (Fig 10). We observed both short [14] and long [11] MTM cycles pattern in the district of Visakhapatnam. The shortest transmission pattern cycle existed for ~ 30 days and longs for ~ 50 days. The incubation period of the parasite shortens dramatically at temperatures in the range of 20°C to 27°C and is responsible for the observed short MTM cycle pattern, whereas the incubation period of the parasite increases in the temperature range of 26°C to 33°C and is responsible for the observed long MTM cycle pattern. In the shortest transmission cycle availability of favorable climatic conditions, infected blood meal, favor the immediate development of mosquitoes as well as the parasites in mosquitoes and the spread of the disease and in longest transmission pattern cycle unavailability of favorable conditions and infected blood meal delay transmission time.

## Conclusion

Climatic conditions in Visakhapatnam district are favourable for the transmission of malaria; the increase in incidences is majorly due to the Climatic factors and disease transmission vectors. The disease incidence is highly influenced by rainfall and monthly average minimum temperature and mosquito population by climatic variables. Multiple regression analysis showed that 27% and 18.3% of the disease incidence and mosquito population respectively are due to climatic conditions. The Multiple regression method is best suited for modeling and prediction of disease incidence and mosquito population and ARIMA (I) model is also suited for prediction of disease incidences. Multiple regression method predicted an increase in malaria disease by 10.98%, 23.85%, 33.47%, and 40.94% for the years 2015, 2020, 2025, 2030 respectively. Developed MTM algorithm identified a major MTM cycle following the June to August rains and occurred between June to September and minor MTM cycles following March to April rains and occurred between March to April were in the District of Visakhapatnam. Fluctuations in climatic factors favored an increase in mosquito populations and thereby increasing the number of malarial cases, rainfall, temperature (20°C to 33°C) and humidity (66% to 81%) maintained a warmer, wetter climate for mosquito growth, parasite development and malaria transmission. Thus, changes in climatic factors influence malaria directly by modifying the behaviour and geographical distribution of vectors and by changing the length of the life cycle of the parasite.

## Supporting Information

S1 Dataset Processed and pooled monthly malaria disease data procured from National Vector Borne Disease Control Programme N.V.D.C.P) Visakhapatnam, India from 43 mandals. (XLS)

S2 Dataset Processed and pooled monthly vector population data procured from National Vector Borne Disease Control Programme (N.V.D.C.P) Visakhapatnam, India (XLS)

S3 Dataset Processed and pooled monthly climatic data procured from Indian Meteriological Centre and Cyclone Warning Centre, Visakhapatnam, India. (XLS)

## References

[1] Kondrachine AV. Malaria in WHO Southeast Asia Region. Indian J Malariol. 1992; 29: 129–160. 1286730

[2] Malaria situation in Andhra Pradesh. Available: http://www.ihs.org.in/HealthSystemsFact Sheets/Malaria.html. Accessed 2014 July 4.

[3] Pavan Kumar STPRC, Reddy NNR. Factors affecting malaria disease transmission and incidence: A special focus on Visakhapatnam district. Int J Rec Sci Res. 2014; 5: 312–317.

[4] Craig MH, Sharp BL, Mabaso ML, Kleinschmidt I. Developing a spatial-statistical model and map of historical malaria prevalence in Botswana using a staged variable selection procedure. Int J Health Geogr. 2007; 24; 6: 44 17892584 10.1186/1476-072X-6-44 PMC2082025

[5] Hay SI, Snow RW. The malaria Atlas Project: developing global maps of malaria risk. PLoS Med. 2006; 3: 12: e473 17147467 10.1371/journal.pmed.0030473 PMC1762059

[6] Hay SI, Guerra CA, Gething PW, Patil AP, Tatem AJ, Noor AM, et al A world malaria map: *Plasmodium falciparum* endemicity in 2007. PLoS Med. 2009; 6: 3: e1000048 10.1371/journal.pmed.1000048 19323591 PMC2659708

[7] Gething PW, Patil AP, Smith DL, Guerra CA, Elyazar IR, Johnston GL, et al A new world malaria map: *Plasmodium falciparum* endemicity in 2010. Malar J. 2011; 10:378 10.1186/1475-2875-10-378 22185615 PMC3274487

[8] World health report Life in 21 st century A vision for all. World Health Organisation, Geneva, Switzerland 226. 1998; Available: http://www.who.int/ whr/1998 /en/w hr98_en.pdf. Accessed 2014 July 4.

[9] Craig MH, Snow RW, le Sueur D. A climate-based distribution model of malaria transmission in sub-Saharan Africa. Parasitol Today. 1999; 15: 105–111. 10322323 10.1016/s0169-4758(99)01396-4

[10] Patz JAGA, McCarty JP, Hussein S, Confalonieri U, de Wet N. Climate change and infectious diseases In: McMichael AJ, Campbell-Lendrum DH, Corvalan CF, Ebi KL, Githeko AK, editors Climate change and humanhealth: risks and responses. Geneva: World Health Organization 2003; pp. 103–132.

[11] Carter R, Mendis KN. Evolutionary and historical aspects of the burden of Malaria. Clin Microbiol Rev. 2002; 15: 564–594. 12364370 10.1128/CMR.15.4.564-594.2002 PMC126857

[12] Rogers DJ, Randolph SE. Climate change and vector-borne diseases. Adv Parasit. 2006; 62: 345–381. 10.1016/S0065-308X(05)62010-6 16647975

[13] Pampana EJ. A textbook of malaria eradication. London: Oxford University Press 1969; 608 p.

[14] Martens WJM, Jetten TH, Rotmans J, Niessen LW. Climate change and vector-borne diseases: a global modelling perspective. Global Environ Chang. 1995; 5:195–209.

[15] Gubler DJ, Reiter PK, Ebi L, Yap W, Nasci R, Patz JA. Climate variability and change in the United States: potential impacts on vector- and rodent-borne diseases. Environ Health Persp. 2001; 109: 223–233. 10.1289/ehp.109-1240669 PMC1240669 11359689

[16] Koenraadt CJM, Majambere S, Hemerik L, Takken W. The effects of food and space on the occurrence of cannibalism and predation among larvae of *Anopheles gambiae* s.1. Entomol Exp Appl. 2004; 112: 125–134.

[17] Krefis AC, Schwarz NG, Nkrumah B, Acquah S, Loag W, Oldeland J, et al Spatial analysis of land cover determinants of malaria incidence in the Ashanti Region, Ghana. PLoS One. 2011; 6:e17905 10.1371/journal.pone.0017905 21448277 PMC3063166

[18] Alemu A, Tsegaye W, Golassa L, Abebe G. Urban malaria and associated risk factors in Jimma town, south-west Ethiopia. Malar J. 2011: 10: 173 10.1186/1475-2875-10-173 21699741 PMC3128012

[19] Woube M. Geographical distribution and dramatic increases in incidences of malaria: consequences of the resettlement scheme in Gambela, SW Ethiopia. Indian J Malariol. 1997; 34: 140–63. 9519570

[20] Musawenkoi LHM, Vounatsou P, Midzi S, Silva J D, Smith T. Spatiotemporal analysis of the role of climate in inter-annual variation of malaria incidence in Zimbabwe. Int J Health Geogr. 2006; 5:20 16700905 10.1186/1476-072X-5-20 PMC1513195

[21] Jones AE, Wort UU, Morse AP, Hastings IM, Gagnon AS. Climate prediction of El Niño malaria epidemics in north-west Tanzania. Malar J. 2007; 6:162 18062817 10.1186/1475-2875-6-162 PMC2228309

[22] Lindsay SW, Bødker R, Malima R, Msangeni HA, Kisinza W. Effect of 1997–98 El Niño on highland malaria in Tanzania. Lancet. 2000; 355: 989–990. 10.1016/s0140-6736(00)90022-9 10768443

[23] Briet OJT, Vounatsou P, Gunawardena DM, Galappaththy GNL, Amerasinghe PH. Models for short term malaria prediction in Sri Lanka. Malar J. 2008; 7: 1–11. 10.1186/1475-2875-7-1 18460204 PMC2412896

[24] Bi Peng, Tong S, Donald K, Parton KA, Ni J. Climatic variables and transmission of malaria: A 12-year data analysis in Shuchen county. Public Health Rep. 2003; 118: 65–71. 12604766 10.1093/phr/118.1.65 PMC1497511

[25] Zhao X, Chen F, Feng Z, Li X, Zhou XH. Characterizing the effect of temperature fluctuation on the incidence of malaria: an epidemiological study in south-west China using the varying coefficient distributed lag non-linear model. Malar J. 2014; 13:192 10.1186/1475-2875-13-192 24886630 PMC4050477

[26] Kilian AH, Langi P, Talisuna A, Kabagambe G. Rainfall pattern, El Niño and malaria in Uganda. Trans R Soc Trop Med Hyg. 1999; 93: 22–23. 10492781 10.1016/s0035-9203(99)90165-7

[27] Mbogo CM, Mwangangi JM, Nzovu J, Gu W, Yan G, Gunter JT, et al Spatial and temporal heterogeneity of *Anopheles* mosquitoes and *Plasmodium falciparum* transmission along the Kenyan coast. Am J Trop Med Hyg. 2003; 68: 734–742. 12887036

[28] Himeidan YE, Hamid EE, Thalib L, Elbashir MI, Adam I. Climatic variables and transmission of falciparum malaria in New Halfa, eastern Sudan. East Mediterr Health J. 2007; 13: 17–24. 17546901

[29] Loevinsohn ME. Climate warming and increased malaria incidence in Rwanda. Lancet. 1994; 343: 714–718. 7907685 10.1016/s0140-6736(94)91586-5

[30] Bouma MJ. Methodological problems and amendments to demonstrate effects of temperature on the epidemiology of malaria. Trans R Soc Trop Med Hyg. 2003; 97: 133–139. 14584363 10.1016/s0035-9203(03)90099-x

[31] Huang F, Zhou S, Zhang S, Wang H, Tang L. Temporal correlation analysis between malaria and meteorological factors in Motuo County, Tibet. Malar J. 2011; 10:54 10.1186/1475-2875-10-54 21375751 PMC3060153

[32] de Souza D, Kelly-Hope L, Lawson B, Wilson M, Boakye D. Environmental factors associated with the distribution of *Anopheles gambiae* s.s in Ghana; an important vector of lymphatic filariasis and malaria. PLoS One. 2010; 5: 3: e9927 10.1371/journal.pone.0009927 20360950 PMC2847902

[33] Devi NP, Jauhari RK. Climatic variables and malaria incidence in Dehradun, Uttaranchal, India. J Vector Borne Dis. 2006; 43: 21–28. 16642782

[34] Baruaha I, Das NG, Kalita J. Seasonal prevalence of malaria vectors in Sonitpur district of Assam, India. J Vector Borne Dis. 2007; 44: 149–153. 17722870

[35] Nath DC, Mwchahary DD. Association between climatic variables and malaria incidence: a study in Kokrajhar district of Assam, India. Glob J Health Sci. 2013; 5: 90–106. 10.5539/gjhs.v5n1p90 PMC4776956 23283041

[36] Bashar K, Tuno N. Seasonal abundance of *Anopheles* mosquitoes and their association with meteorological factors and malaria incidence in Bangladesh. Parasites & Vectors. 2014; 18; 7: 442. 25233890 10.1186/1756-3305-7-442 PMC4262261

[37] Reisen WK, Cayan D, Tyree M, Barker CM, Eldridge B, Dettinger M. Impact of climate variation on mosquito abundance in California. J Vector Ecol. 2008; 33: 1: 89–98. 18697311 10.3376/1081-1710(2008)33[89:iocvom]2.0.co;2

[38] Parham PE, Michael E. Modeling the effects of weather and climate change on malaria transmission. Environ Health Perspect. 2010; 118: 5: 620–6. 10.1289/ehp.0901256 20435552 PMC2866676

[39] Khormi HM. Kumar L. Regression model for predicting adult female *Aedes aegypti* based on meteorological variables: A case study of Jeddah, Saudi Arabia. J Earth Sci Clim Change 2014; 5: 1.

[40] Usher PK. Modelling malaria transmission potential for climate scenarios in West Africa and Europe. Earth Env. 2010; 5: 40–65.

[41] Bomblies A. Modeling the role of rainfall patterns in seasonal malaria transmission Climatic Change. 2012; 112: 3–4: 673–685.

[42] Roiz D, Neteler M, Castellani C, Arnoldi D, Rizzoli A. Climatic factors driving invasion of the tiger mosquito (*Aedes albopictus*) into new areas of Trentino, northern Italy. PLoS One. 2011; 6: 4: e14800 10.1371/journal.pone.0014800 21525991 PMC3078124

[43] Khormi HM, Kumar L. Climate change and the potential global distribution of *Aedes aegypti*: spatial modelling using GIS and CLIMEX. Geospat Health. 2014; 8: 2: 405–15. 24893017 10.4081/gh.2014.29

[44] Rai P.K., Nathawat M.S., and Onagh M. Application of multiple linear regression model through GIS and remote sensing for malaria mapping in Varanasi District, INDIA, Int J Nur Res Rev. 2012; 6: 4: 731–749.

[45] Markham CG. Seasonality of precipitation in the United States. Ann Assoc Am Geogr. 1970; 60: 593–597.

[46] Stuckey EM, Smith T, Chitnis N. Seasonally dependent relationships between indicators of malaria transmission and disease provided by mathematical model simulations. PLoS Comput Biol. 2014; 10: 9: e1003812 10.1371/journal.pcbi.1003812 25187979 PMC4154642

[47] Mabaso ML, Craig M, Vounatsou P, Smith T. Towards empirical description of malaria seasonality in southern Africa: the example of Zimbabwe. Trop Med Int Health. 2005; 10: 9: 909–18. 16135199 10.1111/j.1365-3156.2005.01462.x

[48] Mabaso ML, Craig M, Ross A, Smith T. Environmental predictors of the seasonality of malaria transmission in Africa: the challenge. Am J Trop Med Hyg. 2007; 76: 1: 33–8. 17255225

[49] Childs DZ, Cattadori IM, Suwonkerd W, Prajakwong S, Boots M. Spatiotemporal patterns of malaria incidence in northern Thailand. Trans R Soc Trop Med Hyg. 2006; 100: 623–631. 16406037 10.1016/j.trstmh.2005.09.011

[50] Gemperli A, Sogoba N, Fondjo E, Mabaso M, Bagayoko M, Briët OJ, et al Mapping malaria transmission in West and Central Africa. Trop Med Int Health. 2006; 11: 7:1032–46. 16827704 10.1111/j.1365-3156.2006.01640.x

[51] Grover-Kopec EK, Blumenthal MB, Ceccato P, Dinku T, Omumbo JA, Connor SJ. Web-based climate information resources for malaria control in Africa. Malar J. 2006; 5:38 16689992 10.1186/1475-2875-5-38 PMC1475872

[52] Snow RW, Armstrong-Schellenberg JRM, Peshu N, Forster D, Newton CRJC, Winstanley PA, et al Periodicity and space–time clustering of severe childhood malaria on the coast of Kenya. Trans R Soc Trop Med Hyg. 1993; 87: 386–390. 8249058 10.1016/0035-9203(93)90007-d

[53] Smith T, Charlwood JD, Kihonda J, Mwankusye S, Billingsley P, Meuwissen J, et al Absence of seasonal variation in malaria parasitaemia in an area of intense seasonal transmission. Acta Trop. 1993; 54: 55–72. 8103627 10.1016/0001-706x(93)90068-m

[54] Mpofu SM. Seasonal vector density and disease incidence patterns of malaria in an area of Zimbabwe. Trans R Soc Trop Med Hyg. 1985; 79: 169–175. 4002287 10.1016/0035-9203(85)90327-x

[55] Briet OJT, Vounatsou P, Gunawardena DM, Galappaththy GNL, Amerasinghe PH. Models for short term malaria prediction in Sri Lanka. Malar J. 2008; 7: 1–11. 10.1186/1475-2875-7-1 18460204 PMC2412896

[56] Gething PW, Noor AM, Gikandi PW, Ogara EA, Hay SI, Nixon MS, et al Improving imperfect data from health management information systems in Africa using space time geo-statistics. PLoS Med. 2006; 3: e271 16719557 10.1371/journal.pmed.0030271 PMC1470663

[57] Elliot P, Wakefield J, Best N, Briggs DJ. Spatial epidemiology-methods and applications. Oxford: Oxford University Press 2000 494 p.

[58] Cancre N, Tall A, Rogier C, Faye J, Sarr O, Trape JF, et al Bayesian analysis of an epidemiologic model of *Plasmodium falciparum* malaria infection in Ndiop, Senegal. Am J Epidemiol. 2000; 152: 760–770. 11052555 10.1093/aje/152.8.760

[59] Al-Mansoob MA, Al-Mazzah MM. The role of climate on malaria incidence rate in four governorates of Yemen. Med J Malaysia. 2005; 60: 349–357. 16379191

[60] Tian L, Bi Y, Ho SC, Liu W, Liang S, Goggins WB, et al One-year delayed effect of fog on malaria transmission: a time-series analysis in the rain forest area of Mengla County, south-west China. Malar J. 2008; 7:110 10.1186/1475-2875-7-110 18565224 PMC2441628

[61] Loha E, Lindtjørn B. Model variations in predicting incidence of *Plasmodium falciparum* malaria using 1998–2007 morbidity and meteorological data from south Ethiopia. Malar J. 2010; 9: 166 10.1186/1475-2875-9-166 20553590 PMC2898788

[62] Barnett AG, Dobson AJ. Analysing seasonal health data. London: Springer 2009; 164 p.

[63] Connor SJ, Thomson MC, Flasse SP, Perryman AH. Environmental information systems in malaria risk mapping and epidemic forecasting. Disasters. 1998; 22: 39–56. 9549172 10.1111/1467-7717.00074

[64] Freedman DA. Statistical models: Theory and practice. Cambridge: Cambridge University Press 2005; 414 p.

[65] Ho SL, Xie M. The use of ARIMA models for reliability forecasting and analysis Comput Ind Eng. 1998; 35: 213–216.

[66] Minitab 14 Statistical Software. Computer software. State College, PA: Minitab, Inc 2005 Available: http://www.minitab.com. Accessed 2011 August 27.

[67] World Health Organization. A global strategy for malaria control. Geneva World Health Organization 1993; Available: http://apps.who.int/iris/handle/10665/41785. Accessed 2014 July 4.

[68] Establishing a global partnership to roll back malaria RBM/Draft/1. Geneva: World Health Organization 1998; Available: http://www.rbm.who.int/docs/1gpm/1gpm.pdf. Accessed 2014 July 4.

[69] Connor SJ, Thomson MC, Molyneux DH. Forecasting and prevention of epidemic malaria: new perspectives on an old problem. Parasitologia. 1999; 41: 439–448. 10697900

[70] Githeko AK, Lindsay SW, Confalonieri UE, Patz JA. Climate change and vector-borne diseases: a regional analysis. Bull World Health Organ. 2000; 78: 1136–1147. 11019462 PMC2560843

[71] Thomson M, Indeje M, Connor S, Dilley M, Ward N. Malaria early warning in Kenya and seasonal climate forecasts. Lancet. 2003; 362:580. 10.1016/S0140-6736(03)14135-9 12932403

[72] Thomson MC, Connor SJ. The development of malaria early warning systems for Africa. Trends Parasitol. 2001; 17: 438–445. 11530356 10.1016/s1471-4922(01)02077-3

[73] Fontaine RE, Najjar AE, Prince JS. The 1958 malaria epidemic in Ethiopia. Am J Trop Med Hyg. 1961; 10: 795–803. 13893940 10.4269/ajtmh.1961.10.795

[74] Marimbu J, Ndayiragije A, Le BM, Chaperon J. Environment and malaria in Burundi. Apropos of a malaria epidemic in a non-endemic mountainous region. Bull Soc Pathol Exot. 1993; 86: 399–401. 7819788

[75] Abeku TA, Oortmarssen VGJ, Borsboom G, Vlas DSJ, Habbema JD. Spatial and temporal variations of malaria epidemic risk in Ethiopia: factors involved and implications. Acta Trop. 2003; 87: 331–340. 12875926 10.1016/s0001-706x(03)00123-2

[76] Freeman T, Bradley M. Temperature is predictive of severe malaria years in Zimbabwe. Trans R Soc Trop Med Hyg. 1996; 90: 232 8758057 10.1016/s0035-9203(96)90224-2

[77] Shanks GD, Hay SI, Stern DI, Biomndo K, Snow RW. Meteorologic influences on *Plasmodium falciparum* malaria in the high land tea estates of Kericho, Western Kenya. Emerg Infect Dis. 2002; 8: 1404–1408. 12498655 10.3201/eid0812.020077 PMC2738527

[78] Singh N, Sharma VP. Patterns of rainfall and malaria in Madhya Pradesh, central India. Ann Trop Med Parasitol. 2002; 96: 349–359. 12171616 10.1179/000349802125001113

[79] Musa MI, Shohaimi S, Hashim NR, Krishnarajah I. A climate distribution model of malaria transmission in Sudan. Geospat Health. 2012; 7: 27–36. 23242678 10.4081/gh.2012.102

[80] Grace An, Influence of Climate on Malaria in China Penn McNair Research Journal. 2011; 3: 1.

[81] Beck-Johnson LM, Nelson WA, Paaijmans KP, Read AF, Thomas MB, Bjørnstad ON.The effect of temperature on Anopheles mosquito population dynamics and the potential for malaria transmission. PLoS One. 2013; 8: 11: e79276 10.1371/journal.pone.0079276 24244467 PMC3828393

[82] Blanford JI, Blanford S, Crane RG, Mann ME, Paaijmans KP, Schreiber KV, et al Implications of temperature variation for malaria parasite development across Africa. Sci Rep. 2013; 3:1300 10.1038/srep01300 23419595 PMC3575117

[83] Patz JA, Olson SH. Malaria risk and temperature: influences from global climate change and localland use practices. Proc Natl Acad Sci U S A. 2006; 103: 15: 5635–6. 16595623 10.1073/pnas.0601493103 PMC1458623

[84] Kleinschmidt I, Bagayoko M, Clarke GPY, Craiga M, Sueura DL. A spatial statistical approach to malaria mapping. Int J Epidemiol. 2000; 29: 355–361. 10817136 10.1093/ije/29.2.355

[85] Hay SI, Rogers DJ, Toomer JF, Snow RW. Annual *Plasmodium falciparum* entomological inoculation rates (EIR) across Africa: literature survey, Internet access and review. Trans R Soc Trop Med Hyg. 2000; 94: 113–127. 10897348 10.1016/s0035-9203(00)90246-3 PMC3204456

[86] Gurney WSC, Nisbet RM. Fluctuation periodicity, generation separation, and the expression of larval competition. Theoretical Population Biology. 1985; 28: 150–180.

[87] Pletsch D. Informe sobre una misión efectuada en España en septiembre-noviembre de 1963 destinada a la certificación de la erradicación del paludismo. Revista de Sanidad e Higiene Pública. 1965; 39: 309–367. 5879708

[88] Pachauri, R.K, Reisinger, A. IPCC Fourth Assessment Report (AR4) Contribution of Working Groups I, II and III to the Fourth Assessment Report of the Intergovernmental Panel on Climate Change IPCC, Geneva, Switzerland. 2007; pp 104.

[89] Becker NG. Analysis of infectious disease data. London: Chapman and Hall 1989; 224p.

[90] Daley DJ, Gani J. Epidemic modelling: an introduction. Cambridge: Cambridge University Press 1999 213 p.

